# The p24-family and COPII subunit SEC24C facilitate the clearance of alpha1-antitrypsin Z from the endoplasmic reticulum to lysosomes

**DOI:** 10.1091/mbc.E23-06-0257

**Published:** 2024-02-07

**Authors:** Benjamin S. Roberts, Debashree Mitra, Sudhanshu Abishek, Richa Beher, Prasanna Satpute-Krishnan

**Affiliations:** a Uniformed Services University of the Health Sciences, Bethesda, MD 20814; Nanyang Technological University

## Abstract

A subpopulation of the alpha-1-antitrypsin misfolding Z mutant (ATZ) is cleared from the endoplasmic reticulum (ER) via an ER-to-lysosome-associated degradation (ERLAD) pathway. Here, we report that the COPII subunit SEC24C and the p24-family of proteins facilitate the clearance of ATZ via ERLAD. In addition to the previously reported ERLAD components calnexin and FAM134B, we discovered that ATZ coimmunoprecipitates with the p24-family members TMP21 and TMED9. This contrasts with wild type alpha1-antitrypsin, which did not coimmunoprecipitate with FAM134B, calnexin or the p24-family members. Live-cell imaging revealed that ATZ and the p24-family members traffic together from the ER to lysosomes. Using chemical inhibitors to block ER exit or autophagy, we demonstrated that p24-family members and ATZ co-accumulate at SEC24C marked ER-exit sites or in ER-derived compartments, respectively. Furthermore, depletion of SEC24C, TMP21, or TMED9 inhibited lysosomal trafficking of ATZ and resulted in the increase of intracellular ATZ levels. Conversely, overexpression of these p24-family members resulted in the reduction of ATZ levels. Intriguingly, the p24-family members coimmunoprecipitate with ATZ, FAM134B, and SEC24C. Thus, we propose a model in which the p24-family functions in an adaptor complex linking SEC24C with the ERLAD machinery for the clearance of ATZ.

## Abstract (executive summary)

The intracellular accumulation of the Z mutant of the secretory protein, alpha-1-antitrypsin (ATZ), leads to liver cell death. To prevent accumulation, a subpopulation of ATZ is cleared from the endoplasmic reticulum (ER) via a FAM134B-dependent, ER-to-lysosome-associated-degradation (ERLAD) pathway.

How ATZ is captured into ER-exit sites for ERLAD is unknown.

Combined imaging and biochemical approaches reveal that the p24-family proteins, TMED9 and TMP21, interact with ATZ, FAM134B and SEC24C, and that depletion of TMED9, TMP21 or SEC24C blocks ATZ trafficking to lysosomes. These findings demonstrate the involvement of p24-family proteins and SEC24C in promoting the clearance of ATZ via ERLAD.

## INTRODUCTION

In eukaryotic cells, approximately one-third of the total proteome is synthesized and folded in the endoplasmic reticulum (ER) before transport along the secretory pathway (Braakman and Bulleid, 2011). Because protein folding is an error-prone process (Dobson, 2003; Princiotta *et al.*, 2003), ER protein quality control (PQC) systems monitor folding and redirect misfolded proteins for degradation ( Adams *et al.*, 2019; Sun and Brodsky, 2019). The best studied ER clearance pathways are collectively called ER associated degradation (ERAD) and involve retrotranslocation of soluble and membrane-bound proteins across the ER membrane into the cytosol for proteasomal degradation (Vashist and Ng, 2004; Krshnan *et al.*, 2022). Nonetheless, as the fates of increasingly diverse secretory pathway substrates with different topologies and misfolding characteristics have been delineated, multiple distinct vesicular ER-clearance pathways have been uncovered. For example, misfolded glycosylphosphatidylinositol (GPI)-anchored proteins (GPI-APs) are cleared by a p24-family dependent ER-to-Golgi vesicular pathway called rapid ER stress-induced export (RESET) that culminates in lysosomal degradation (Satpute-Krishnan *et al.*, 2014). Additionally, diverse aggregation-prone secretory proteins are cleared from ER to lysosomes by various vesicular pathways recently classified by Fregno, Molinari and colleagues under the umbrella term “ER-to-lysosome-associated-degradation” (ERLAD; Omari *et al.*, 2018; Cui *et al.*, 2019; De Leonibus *et al.*, 2019; Forrester *et al.*, 2019; Fregno and Molinari, 2019; Oikonomou and Hendershot, 2020; Wilkinson, 2020; Gorrell *et al.*, 2021).

The natural propensity of proteins to misfold, genetic mutations, and limitations or defects in the existing PQC systems can lead to the accumulation of misfolded proteins within cells, often producing toxic gain-of-function effects that can lead to cell death and disease in the organism (Winklhofer *et al.*, 2008; Silverman *et al.*, 2013). A particularly notable example of this is the Glu342Lys point mutation of the serine protease inhibitor alpha-1-antitrypsin (AAT), called alpha-1-antitrypsin Z allele (ATZ). Normally, the liver produces and releases AAT into circulation where it protects tissues from proteases including neutrophil elastase (Perlmutter, 2016; Janciauskiene *et al.*, 2018). ATZ not only fails to perform its protective function, but also misfolds and forms toxic aggregates that contribute to liver disease (Lomas *et al.*, 1992; Strnad *et al.*, 2020). Studies into the ATZ trafficking pathways reveal that while a fraction of ATZ appears to be cleared by ERAD (Qu *et al.*, 1996; Teckman *et al.*, 2001; Perlmutter, 2011), the remainder accumulates intracellularly in ER-derived inclusion bodies marked with ER resident proteins (Granell and Baldini, 2008; Granell *et al.*, 2008) before undergoing autophagy-dependent degradation (Teckman and Perlmutter, 2000; Perlmutter, 2011; Feng *et al.*, 2017). Enhancing autophagy appears to promote clearance of ATZ aggregates (Hidvegi *et al.*, 2010; Kaushal *et al.*, 2010).

Recently, Fregno, Molinari, and colleagues (2018) dissected a detailed pathway for misfolded, aggregated ATZ that involves the release of ATZ from the ER-resident chaperone, calnexin, into FAM134B-populated ER-derived vesicles for direct ER-to-lysosome delivery. They demonstrated that this pathway requires the LC3-interacting region of FAM134B for delivery of ATZ into LC3-II displaying lysosomes and soluble N-ethylmaleimide-sensitive factor attachment protein receptors (SNAREs) dependent fusion (Fregno *et al.*, 2018). Intriguingly, Cui, Ferro-Novick and colleagues (2019) demonstrated that FAM134B requires the coat protein complex II (COPII) subunit SEC24C, but not other SEC24 isoforms, for ER to lysosome transport in mammalian cells, and that this interaction was conserved in yeast. It is well-established that COPII operates at ER exit sites during vesicle formation for the anterograde transport of secretory pathway proteins from the ER to the Golgi (Zanetti *et al.*, 2011). Recent studies revealed a new role for COPII in LC3-mediated ER-to-lysosomal delivery pathways for misfolded aggregated secretory cargos such as procollagen I (Omari *et al.*, 2018; Gorrell *et al.*, 2021), the proarginine-vasopressin variant Gly57Ser, the proopiomelanocortin variant Cys28Phe, and the proinsulin mutant *Akita* (Cui *et al.*, 2019; Parashar *et al.*, 2021). These new findings pose the question: how are misfolded proteins recruited to and packaged into ER-derived vesicles for direct transport to lysosomes?

Apart from calnexin and FAM134B, little is known about which partner proteins contribute to the packaging and export of the misfolded proteins in ER-derived ERLAD vesicles. FAM134B displays no known COPII binding signature domains in the cytosol and lacks extensive luminal domains to associate with misfolded proteins in the ER lumen. Calnexin interacts with both FAM134B and misfolded ATZ, however it does not enter ERLAD vesicles (Fregno *et al.*, 2018, 2021). Thus, FAM134B may rely on other factors to associate with SEC24C and to coordinate the delivery of misfolded proteins to lysosomes. Because the p24-family of proteins are well established as COPII binding proteins, and TMP21 and TMED9 associate with misfolding proteins including GPI-APs (Satpute-Krishnan *et al.*, 2014; Zavodszky and Hegde, 2019) and mucin-1 mutants (Dvela-Levitt *et al.*, 2019), we hypothesized that the p24-family proteins may act as the COPII receptor involved in ATZ clearance via ERLAD.

Here, for the first time, we have identified a requisite role for SEC24C and the p24-family COPII-binding proteins, TMP21 (also known as TMED10 or p24δ1) and TMED9 (also known as p24α2 or p25) in the clearance of ATZ. We examined the interactions between the p24-family, SEC24C, FAM134B, and ATZ that may contribute to our understanding of how cells package misfolded proteins in the ER for lysosomal delivery and provide insights to help combat toxic gain-of-function proteinopathies.

## RESULTS

### ERLAD substrate, ATZ, associates with p24-family members during steady-state conditions

The mechanism by which ATZ is packaged into ER exit sites destined for transport to lysosomes via ERLAD is not well understood. To gain insight into this process, we selected mouse Neuro-2a (N2a) cells and normal rat kidney (NRK) cells as model systems for the following reasons. N2a cells are ideal for biochemical analysis of ATZ trafficking because they are amenable to transfection and were used in previous ATZ studies (Granell *et al.*, 2008). However, N2a cells are highly mobile and have low adherence to the coverslip, making it very difficult to trace the trafficking pathways of proteins in live cells by time-lapse imaging (Cheatham *et al.*, 2023). NRK cells have a low transfection efficiency of ∼10%, but they are ideal for long imaging experiments because they are relatively immobile and tightly adherent to the coverslip and, from our experience, preserve the secretory pathway protein trafficking, quality control, and degradation pathways that we have studied in N2a, HeLa, and other cell culture types (Hailey *et al.*, 2010; Satpute-Krishnan *et al.*, 2014; Sengupta *et al.*, 2015; Cheatham *et al.*, 2023). Furthermore, the NRK cell line we use here demonstrates reversible inhibition of ER-export with brefeldin A (Lippincott-Schwartz *et al.*, 1989, 1990; Donaldson *et al.*, 1990, 1991).

We generated a panel of expression constructs encoding carboxy (C)-terminally or amino (N)-terminally tagged variants of the wild type AAT and mutant ATZ. These included AAT-Venus (VEN), AAT-Cerulean (CER), ATZ-VEN, VEN-AAT, VEN-ATZ, SNAP-FLAG-AAT (“S-FLAG-AAT”) and SNAP-FLAG-ATZ (“S-FLAG-ATZ”). Specifically for all of the N-terminally tagged variants, we opted to use the rabbit lactase phlorizin-hydrolase lactase signal sequence “SS(lactase)” ahead of the N-terminal tag because it efficiently drives translocation into the ER. This allowed cells to express a homogenous pool of ATZ that translocated into the ER and reduced the likelihood of a second cytosolic population. The efficiency of SS(lactase) was revealed through early pulse-chase and Endo H glycosylation studies where lactase initially appeared as a single uniform population Endo H sensitive nascent protein that eventually became complex glycosylated and processed (Hauri *et al.*, 1985; Naim *et al.*, 1987; Mantei *et al.*, 1988; Ouwendijk *et al.*, 1998). Additionally, SS(lactase) has been used previously for efficient translocation of GFP-tagged proteins into the ER (Keller *et al.*, 2001; Satpute-Krishnan *et al.*, 2014).

**FIGURE 1:**
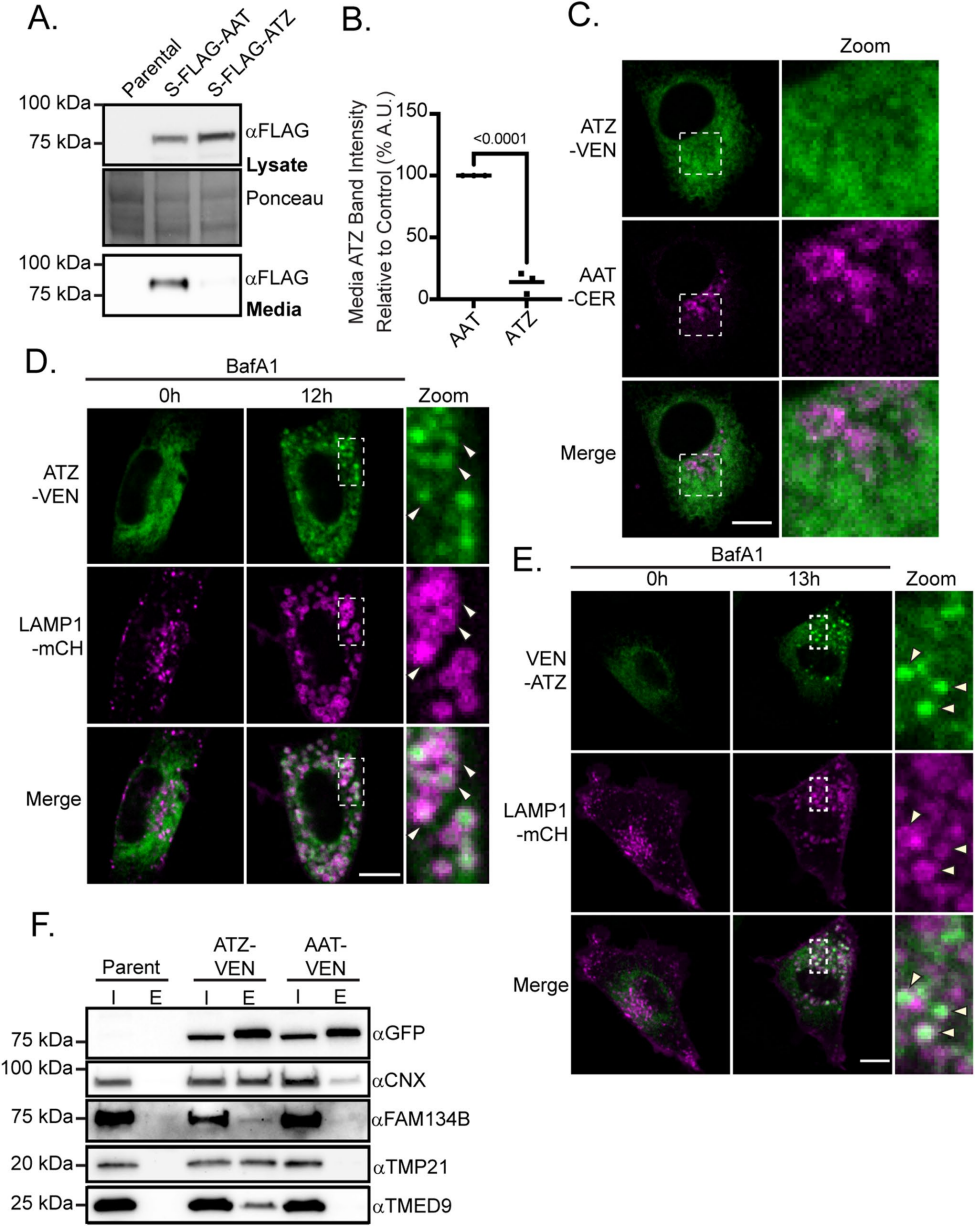
ERLAD substrate, ATZ, associates with p24-family members during steady-state conditions. (A) Immunoblots of cell lysates and culture media from S-FLAG-AAT or S-FLAG-ATZ expressing N2a cells that were probed with FLAG antibody. Total cellular protein was probed with Ponceau S. Similar results were obtained in each replicate experiment. (B) Plot of the intensity of the FLAG band for S-FLAG-ATZ (*n* = 3, mean = 14 ± 8%) in culture medium relative to the FLAG band for S-FLAG-AAT (*n* = 3), line represents mean. *P* value calculated with an unpaired *t* test with Welch’s correction. (C) Representative confocal image of NRK cells coexpressing ATZ-VEN and AAT-CER. (D) Time-lapse images of a representative NRK cell coexpressing ATZ-VEN and LAMP1-mCH and treated with 250 nM bafilomycin A1 (BafA1, see Supplemental Video 1). (E) Time-lapse images of a representative NRK cell coexpressing VEN-ATZ and LAMP1-mCH and treated with 250 nM BafA1 (see Supplemental Video 2). (F) Immunoblots of GFP-tag co-immunoprecipitations from untransfected N2a cells (parent) or N2a cells expressing ATZ-VEN or AAT-VEN. In addition to GFP to detect coimmunoprecipitation of VEN-tagged constructs, blots were probed for calnexin (CNX), FAM134B, TMP21, and TMED9. The experiments shown in panels (A–F) were repeated in three or more biological replicates. Scale bars = 10 μm.

The fates of wild type AAT, which is secreted, and mutant ATZ, which is less efficiently secreted and instead partially retained intracellularly and routed from the ER to lysosomes for degradation, have been long established in cell culture and animal models (Carrell *et al.*, 1982; Gross *et al.*, 1982; Carlson *et al.*, 1989; Le *et al.*, 1990; Lomas *et al.*, 1992). To dissect the quality control mechanisms that regulate the clearance of ATZ from the ER to lysosomes, we first confirmed that we could replicate these earlier findings using our tagged substrates. First, we confirmed that S-FLAG-ATZ is less efficiently secreted into the medium in comparison to the S-FLAG-AAT (Figure 1, A and B). Second, we confirmed that during steady-state conditions intracellular ATZ-VEN is localized in a large internal compartment that is consistent with the ER, while intracellular AAT-CER primarily occupies the perinuclear region, consistent with a Golgi localization (Figure 1C), as demonstrated previously (Granell *et al.*, 2008). Replication of these phenotypes in our cell culture systems indicated that the AAT and ATZ trafficking pathways are largely universal. We next sought to confirm that ATZ underwent ERLAD in NRK cells regardless of the tag placement. To confirm this, we examined the trafficking of both C-terminally tagged ATZ-VEN and N-terminally tagged VEN-ATZ to lysosomes. Both VEN-ATZ and ATZ-VEN accumulated in the lysosomes of NRK cells that were treated with the vacuolar-type ATPase inhibitor bafilomycin A1 (Figure 1, D and E; Supplemental Videos 1 and 2), (Fregno *et al.*, 2018, 2021), demonstrating that ATZ tagged at either the N or C terminal undergoes ERLAD.

**Figure:** Movie S1 ATZ‐VEN undergoes ERLAD in NRK cells. NRK cells were co‐transfected with ATZ‐VEN (green) and LAMP1‐mCH (magenta) and imaged over 12 hours after treatment with bafilomycin A1.

**Figure:** Movie S2 VEN‐ATZ undergoes ERLAD in NRK cells. NRK cells were co‐transfected with VEN‐ATZ (green) and LAMP1‐mCH (magenta) and imaged over 12 hours after treatment with bafilomycin A1.

TMP21 and other p24-family members associate with and are required for the clearance of a subclass of misfolded GPI-APs from the ER via the RESET pathway (Satpute-Krishnan *et al.*, 2014; Zavodszky and Hegde, 2019). The p24 proteins interact with misfolded GPI-APs that are retained within the ER lumen. Because ATZ is also a misfolded protein that is retained within the ER, we investigated whether ATZ interacts with p24 family members. We column purified ATZ-VEN with antibodies against the VEN-tag under nondenaturing conditions and probed the eluates for TMP21. Intriguingly, ATZ-VEN copurified with TMP21, albeit less efficiently than the positive control YFP-PrP* (Figure 1F; Supplemental Figure S1A). ATZ-VEN also copurified with another p24 family member, TMED9 (Figure 1F). We additionally confirmed that ATZ interacts with calnexin and FAM134B (Figure 1F), as was previously reported (Fregno *et al.*, 2018). Critically, TMP21, TMED9, calnexin, and FAM134B associated with mutant ATZ-VEN more strongly than with wild type AAT-VEN (Figure 1F), suggesting a role for these factors in PQC of ATZ. Because the p24-family members were previously shown to be involved in RESET, we checked whether a fraction of the ATZ-VEN population undergoes RESET by coexpressing ATZ-VEN with CFP-PrP* and treating cells with the ER stressor thapsigargin, which triggers the synchronous release of RESET substrates for ER-export (Satpute-Krishnan *et al.*, 2014; Zavodszky and Hegde, 2019). While the majority of CFP-PrP* trafficked from the ER to the Golgi within 30 min, ATZ-VEN remained in the ER (Supplemental Figure S1, B and C). Thus, although TMP21 and TMED9 are required for RESET of PrP* (Satpute-Krishnan *et al.*, 2014; Zavodszky and Hegde, 2019), their association with ATZ does not drive them to lysosomes via the RESET pathway. We next set out to understand the role of TMP21 and other p24 family members in the trafficking of ATZ.

### SEC24C is essential for the ER-to-lysosome-associated degradation of ATZ

The p24-family of proteins are well established as COPII assembling proteins that bind, in particular, to mammalian SEC24C or its yeast homolog LST1 (Dominguez *et al.*, 1998; Tang *et al.*, 1999; Miller *et al.*, 2003; Bonnon *et al.*, 2010; Castillon *et al.*, 2011; D’Arcangelo *et al.*, 2015). Intriguingly, using mammalian U2OS cells, Cui *et al.* (2019) demonstrated that SEC24C is required for the lysosomal degradation of FAM134B, a requisite factor for ERLAD of ATZ (Fregno *et al.*, 2018). Additionally, Cui *et al.* (2019) demonstrated that LST1 is required for the ER clearance of ATZ in yeast. To explain this, they proposed a new role for COPII in specifying ER domains for delivery to lysosomes. Furthermore, TMED9 was recently shown to participate in the biogenesis of autophagosomal membranes at ER exit sites (Li *et al.*, 2022). Therefore, we hypothesized that the p24 family proteins were required for the recruitment of SEC24C proteins to ER exit sites where ERLAD is occurring.

**FIGURE 2:**
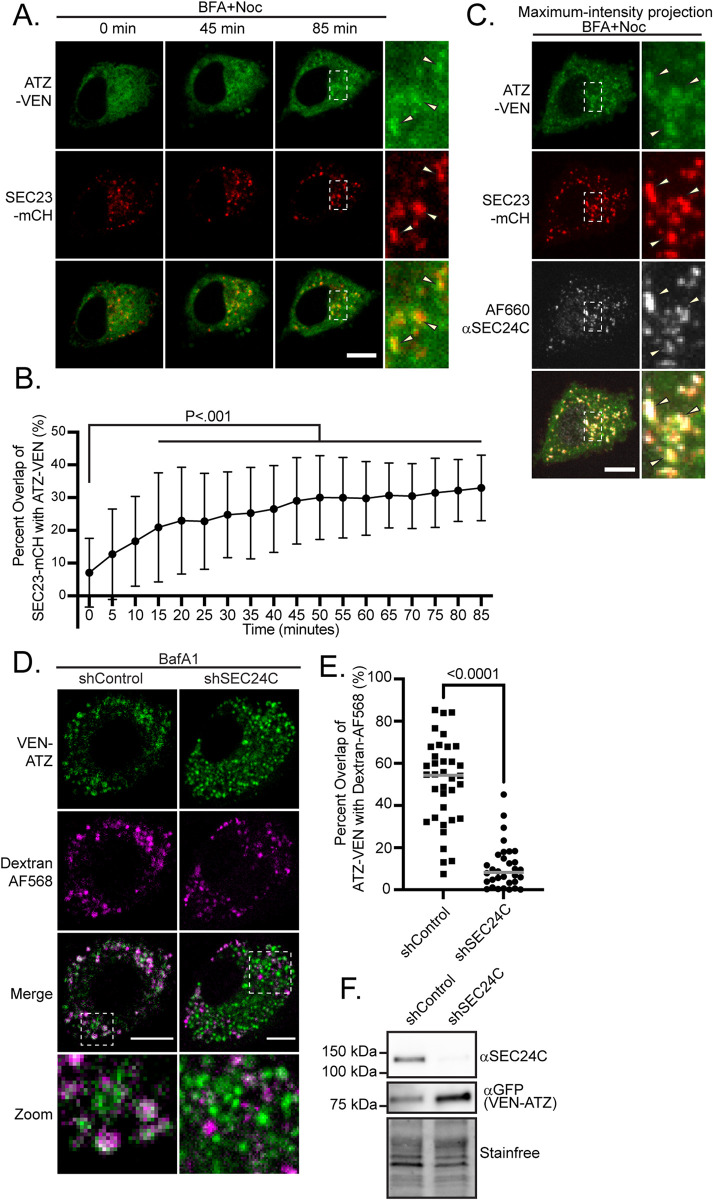
SEC24C is essential or the ERLAD of ATZ. (A) A representative NRK cell expressing ATZ-VEN and SEC23-mCH treated with BFA+Noc and imaged live for 85 min. Arrows indicate sites of ATZ-SEC23 overlap (see Supplemental Videos 3 and 4). (B) The Mander’s overlap coefficient for SEC23-mCH with ATZ-VEN was measured for each time point during live imaging. The percentage of SEC23-mCH overlapping ATZ-VEN is plotted for 31 cells treated with BFA+Noc. *P* value was calculated with a one-way ANOVA with Dunnett’s post-hoc test. Error bars indicate SD from the mean. (C) After 85 min of imaging, the cell shown in (A) was fixed and stained for SEC24C (AF660-αSEC24C). A maximum-intensity Z-projection spanning 4.2 μm is shown. Arrows indicate sites of ATZ-SEC24C overlap. (D) NRK cells were stably-transfected with VEN-ATZ and either shControl or shSEC24C. Cells were preloaded with dextran (dextran-AF568) to mark lysosomes before being treated with BafA1 for 16 h. Cells were subsequently fixed and imaged for colocalization analysis. Arrowheads point to ATZ puncta that colocalizing with the dextran. (E) The Mander’s overlap coefficient for VEN-ATZ with dextran was measured for individual cells from each of three replicate experiments. The percentage of VEN-ATZ overlapping dextran is plotted for shControl cells (*n* = 36, mean = 51 ± 20%) and shSEC24C cells (*n* = 32, mean = 11 ± 11%), line represents median. *P* values were calculated with the Mann-Whitney test. (F) Stable cells were lysed and samples were analyzed by immunoblot for SEC24C and GFP (ATZ-VEN). Similar results were obtained in each replicate experiment. The experiments shown in panels (A–E) were repeated in three or more biological replicates The experiment shown in panel (F) was repeated twice. Scale bars = 10 μm.

We first asked whether SEC24C could play a role in the packaging of ATZ in mammalian cells. Our first step was to assess the colocalization of ATZ-VEN with SEC24C at SEC23-mCherry (mCH) marked ER exit sites. We treated NRK cells coexpressing ATZ-VEN and SEC23-mCH with a combination of brefeldin A and nocodazole (BFA + Noc) to trap secretory proteins at ER exit sites as previously described (Dukhovny *et al.*, 2008; Shomron *et al.*, 2021), and performed live cell time-lapse imaging (Figure 2A; Supplemental Video 3). We observed a significant increase in the proportion of ATZ-VEN overlapping with SEC23-mCH puncta (Figure 2B, *n* = 31). For the representative cell shown in Figure 2A, we fixed and stained it for SEC24C immediately after live-cell imaging and reimaged it; ATZ/SEC23 puncta were marked by or adjacent to SEC24C (Figure 2C). We confirmed that an N-terminal VEN tag on ATZ did not interrupt this process (Supplemental Video 4).

**Figure:** Movie S3 ATZ‐VEN accumulates with SEC23‐mCH during treatment with brefeldin A and nocodazole. NRK cells were co‐transfected with ATZ‐VEN (green) and SEC23‐mCH (magenta). Cells were treated with BFA+Noc and imaged at 5‐minute intervals for up to 90 minutes.

**Figure:** Movie S4 VEN‐ATZ accumulates with SEC23‐mCH during treatment with brefeldin A and nocodazole. NRK cells were co‐transfected with VEN‐ATZ (green) and SEC23‐mCH (magenta). Cells were treated with BFA+Noc and imaged at 5‐minute intervals for up to 90 minutes.

We next examined whether SEC24C is involved in the clearance of ATZ from the ER for lysosomal degradation by examining the impact of SEC24C depletion on ATZ trafficking to lysosomes. We preloaded NRK cells stably expressing VEN-ATZ and either a control short hairpin RNA (shRNA, shControl) or a shRNA targeting SEC24C (shSEC24C) with fluorescently-conjugated dextran beads that ultimately traffic to lysosomes (Humphries *et al.*, 2011). We then analyzed the colocalization of VEN-ATZ with dextran-marked lysosomes after 12 h of treatment with bafilomycin A1. Compared to control cells (*n* = 36, mean = 51 ± 20%), cells expressing shSEC24C (*n* = 32, mean = 11 ± 11%) showed a reduction in ATZ colocalizing with dextran (Figure 2, D and E), implying a role for SEC24C machinery in the delivery of ATZ to lysosomes. SEC24C knockdown was confirmed by immunoblot (Figure 2F).

The requirement of SEC24C for the efficient delivery of ATZ to lysosomes (Figure 2, D and E; Cui *et al.*, 2019) suggests the possibility that the formation of the COPII coat is an integral step in the ERLAD pathway. While FAM134B is required for ATZ clearance (Fregno *et al.*, 2018), prior analysis of FAM134B indicates that it does not have a predicted or demonstrated COPII binding signal (Bhaskara *et al.*, 2019; Mo *et al.*, 2020; Reggio *et al.*, 2021), leaving open the question of how FAM134B, ATZ, and SEC24C are linked together. We hypothesized that p24 family members could serve as adaptors to recruit SEC24C to ER exit sites where ERLAD is occurring based on the following pieces of evidence derived from mammalian cell systems: (1) p24-family has been shown to copurify with ATZ (Figure 1F), as well as other misfolded proteins (Satpute-Krishnan *et al.*, 2014; Dvela-Levitt *et al.*, 2019), and (2) p24 family members are known to copurify or cofractionate with COPII coat components including SEC24C (Dominguez *et al.*, 1998; Bonnon *et al.*, 2010).

### P24-family members assemble with ATZ at ERLAD exit sites and cotraffic to lysosomes

We next sought to explore the role of the p24 proteins in ERLAD. Unfortunately, commercially available antibodies yielded inadequate immunofluorescence results. Thus, we opted to use fluorescently tagged p24 proteins in subsequent imaging experiments. A body of literature shows that the expression of p24-family member constructs (i.e., p24 [also known as TMED2], TMED9 or TMP21) encoding green fluorescent protein (GFP) or GFP variant tags inserted between the N-terminal signal sequence and the luminal domain each produce N-terminally tagged proteins that retain native protein trafficking dynamics and protein–protein interactions (Blum *et al.*, 1999, 2000; Barr *et al.*, 2001; Majoul *et al.*, 2001; Gupta and Swarup, 2006; Simpson *et al.*, 2006; Blum and Lepier, 2008). Thus, we generated N-terminally-tagged constructs comprised of a signal sequence, CER fluorescent protein, and either TMP21 (CER-TMP21) or TMED9 (CER-TMED9) for use in protein interaction studies and live cell imaging.

**FIGURE 3:**
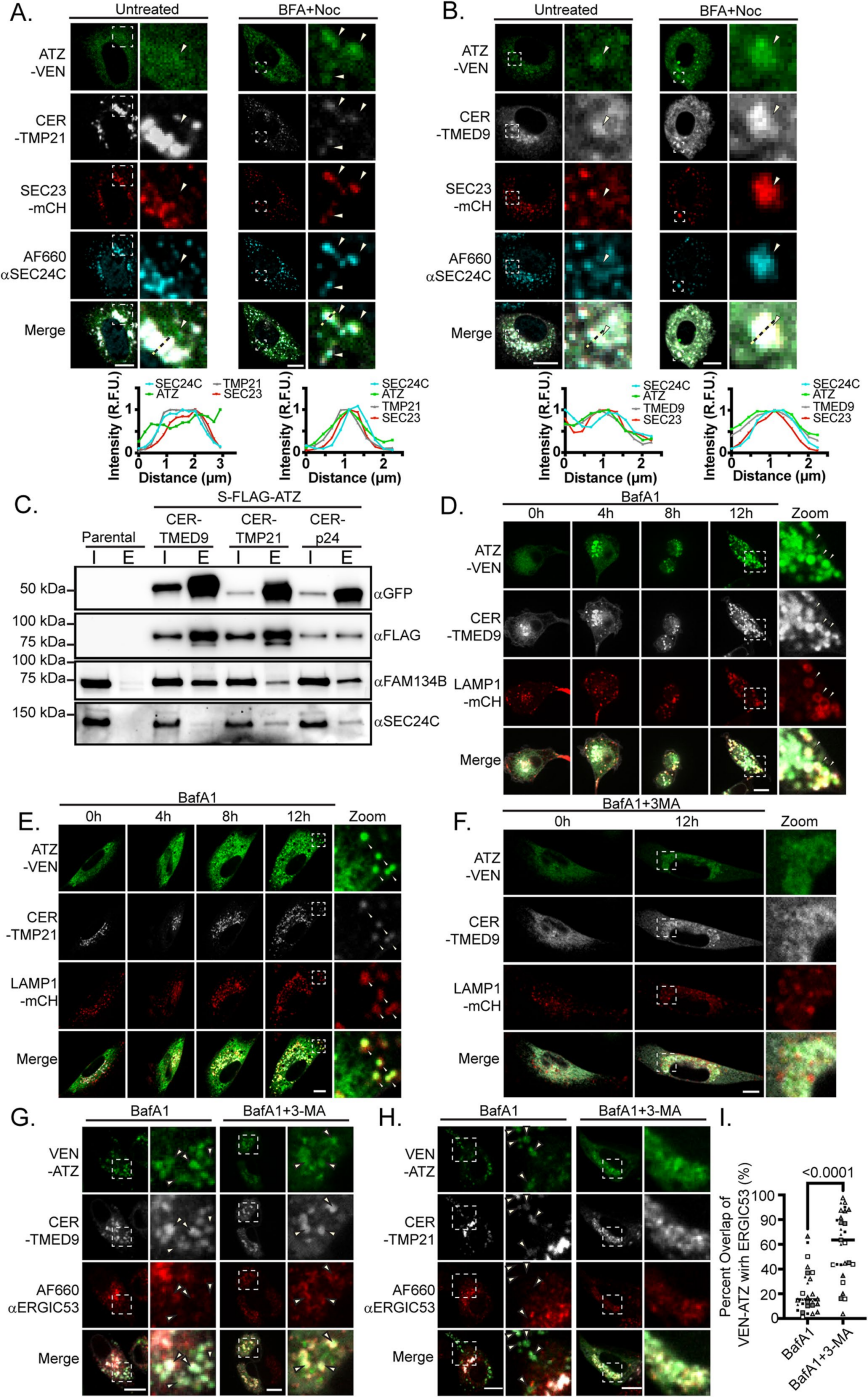
p24 family members assemble with ATZ, SEC24C, and FAM134B at ERLAD exit sites and cotraffic with ATZ to lysosomes. (A) Immunofluorescence of SEC24C (AF660-αSEC24C) in NRK cells coexpressing ATZ-VEN, CER-TMP21, and SEC23-mCH that were untreated or treated with BFA+Noc. Zoom boxes show representative areas of concentrated ATZ expression. Arrows indicate areas of ATZ accumulation. Beneath each of the zoom boxes is a fluorescence intensity plot that correlates with the dotted line drawn within the above zoom box. Fluorescence intensity relative to the lowest intensity value was plotted for each channel across the dotted line. (B) As in (A), except that CER-TMED9 replaces CER-TMP21. (C) Immunoblots of CER-tag coimmunoprecipitations from untransfected N2a cells (parent) or N2a cells coexpressing S-FLAG-ATZ and either CER-TMED9, CER-TMP21, or CER-p24 were analyzed by immunoblot for GFP, FLAG, FAM134B, and SEC24C. Experiment is representative of a single experiment (*n* = 1). (D) NRK cells expressing ATZ-VEN, CER-TMED9, and Lamp1-mCH were treated with 250 nM BafA1 and live-imaged for 12 h. At 12 h (12 h), ATZ and TMED9 puncta colocalize within Lamp1-marked vesicles (Zoom, arrows) (see Supplemental Video 5). (E) As in (D) except that CER-TMP21 replaces CER-TMED9. After cell division at 8 h (8 h), one daughter cell is presented at 12 h (12 h) (see Supplemental Video 6). (F) NRK cells expressing ATZ-VEN, CER-TMED9, and Lamp1-mCH were treated with 250 nM BafA1 and 10 mM 3-methyladenine and live-imaged for 12 h. After 12 h, ATZ and TMED9 colocalize in a perinuclear structure excluded from Lamp1-marked vesicles (see Supplemental Video 8). (G) NRK cells expressing VEN-ATZ and CER-TMED9 were treated with either BafA1 or bafilomycin A1 + 3-methyladenine overnight. Cells were fixed and stained for ERGIC53. Arrows indicate sites of ATZ colocalizing with TMED9. (H) As in (G) except that cells are coexpressing CER-TMP21. (I) The Mander’s overlap coefficient for VEN-ATZ with ERGIC53 was measured for individual cells. The percentage of ATZ overlapping ERGIC53 is plotted for (G) and (H) in cells treated with BafA1 (*n* = 28, mean = 21 ± 18%) or 3-methyladenine + bafilomycin A1 (“BafA1+3-MA”) (*n* = 29, mean = 60 ± 27%), line represents median. Each shape (cells coexpressing CER-TMP21: ▴, cells co-expressing CER-TMED9:◾▫) is colored based on the biological set it came from. *P* value was calculated with a Mann-Whitney test. Similar results were obtained in each replicate experiment. The experiments shown in panels (A–I) were repeated in three or more biological replicates. Scale bars = 10 μm.

To determine whether the p24-family members play a role in the clearance of ATZ via ERLAD, we first tested whether they colocalize with ATZ at sites marked by SEC24C and SEC23-mCH. During steady-state conditions, we again observed that ATZ-VEN did not readily accumulate at ER exit sites marked with SEC23-mCH and SEC24C (Figure 3, A and B [left panels labeled “Untreated”]). To verify that ER-localized ATZ colocalized with SEC23-mCH, we again trapped ATZ-VEN at ER-exit sites using BFA + Noc. As before, we observed greater overlap of ATZ-VEN puncta with SEC23-mCH and SEC24C in cells treated with BFA + Noc (Figure 3, A and B [right panels labeled “BFA + Noc”]). Furthermore, these ATZ-VEN and SEC23-mCH enriched puncta colocalized with p24 family members, CER-TMP21 (Figure 3A) and CER-TMED9 (Figure 3B).

**Figure:** Movie S5 ATZ and TMED9 co‐traffic to lysosomes. NRK cells were co‐transfected with ATZ‐VEN (green), CER‐TMED9 (gray), and LAMP1‐mCH (red) and imaged over 12 hours after treatment with bafilomycin A1.

**Figure:** Movie S6 ATZ and TMP21 co‐traffic to lysosomes. NRK cells were co‐transfected with ATZ‐VEN (green), CER‐TMP21 (gray), and LAMP1‐mCH (red) and imaged over 12 hours after treatment with bafilomycin A1.

To test whether p24-family members associate with ATZ, SEC24C, and the ERLAD machinery FAM134B and calnexin at steady-state, we probed for these protein–protein interactions using pulldowns. We coexpressed S-FLAG-ATZ with one of three CER-tagged p24-family members, TMP21, TMED9, or p24, in N2a cells and pulled down the CER-tagged p24-family members using anti-GFP antibodies. Each of the three CER-tagged p24 family members copurified with S-FLAG-ATZ (Figure 3C). Importantly, we found that CER-tagged TMP21, TMED9, and p24 each copurified with FAM134B and SEC24C as well (Figure 3C). Intriguingly, the associations between FAM134B and TMP21, TMED9 and p24 were previously published in large scale affinity-purification mass spectrometry supplemental datasets of FAM134B coimmunoprecipitates (Grumati *et al.*, 2017; Gonzalez *et al.*, 2023).

Next, we tested whether ATZ-VEN accumulates in LAMP1-mCH marked lysosomes together with CER-TMP21 or CER-TMED9 using time lapse imaging of cells treated with bafilomycin A1. In NRK cells treated with bafilomycin A1, ATZ-VEN accumulated in lysosomes over the course of 8 to 12 h (Figure 3, D and E; Supplemental Videos 5 and 6). We observed the same results in HeLa cells (Supplemental Figure S2; Supplemental Video 7), indicating that lysosomal trafficking of ATZ with p24-family members is a cell type-independent process.

**Figure:** Movie S7 ATZ and TMP21 co‐traffic to lysosomes in HeLa cells. HeLa cells were co‐transfected with ATZ‐VEN (green), CER‐TMP21 (gray), and LAMP1‐mCH (red) and imaged over 12 hours after treatment with bafilomycin A1.

We next sought to verify that p24-family members traffic together with ATZ to lysosomes along the ERLAD pathway instead of arriving by a separate pathway. We tested this by blocking the ERLAD pathway and used confocal imaging to see whether p24 and ATZ were trapped together in a prelysosomal compartment. Because ER clearance of ATZ to lysosomes relies on the binding of FAM134B to LC3-II displayed on the surface of lysosomes (Fregno *et al.*, 2018), we used 3-methyladenine, a class III PI3 kinase inhibitor that blocks upstream events preceding the formation of LC3-II (Petiot *et al.*, 2000), to block ERLAD. Unlike in cells treated with bafilomycin A1 alone (Figure 3, D and E), in cells coincubated with bafilomycin A1 and 3-methyladenine, ATZ-VEN did not accumulate in lysosomes but instead accumulated in perinuclear structures marked with CER-TMED9 (Figure 3F; Supplemental Video 8). To determine whether these perinuclear compartments were ER-derived, we performed immunofluorescence of ERGIC53. In cells coexpressing ATZ-VEN and either CER-TMP21 or CER-TMED9, significantly more ATZ-VEN colocalized with ERGIC53 in cells coincubated with 3-methyladenine and bafilomycin A1 (mean = 60 ± 27% per cell from a combination of cells expressing TMP21 and TMED9 [*n* = 29]) than in cells treated with bafilomycin A1 alone (mean = 21 ± 18% per cell from a combination of cells expressing TMP21 and TMED9 (*n* = 28; Figure 3, G and H, quantified in Figure 3I). These data demonstrate that ATZ-VEN and p24-family members including CER-TMED9 or CER-TMP21, traffic together during ERLAD.

**Figure:** Movie S8 3‐methyladenine prevents ATZ and TMED9 from trafficking from the ER to lysosomes and reroutes them into an alternate ER‐derived compartment. NRK cells were co‐transfected with ATZ‐VEN (green), CER‐TMED9 (gray), and LAMP1‐mCH (red) and imaged over 12 hours after treatment with bafilomycin A1 and 3‐methyladenine.

### p24-family proteins are critical for lysosomal trafficking of ATZ

**FIGURE 4:**
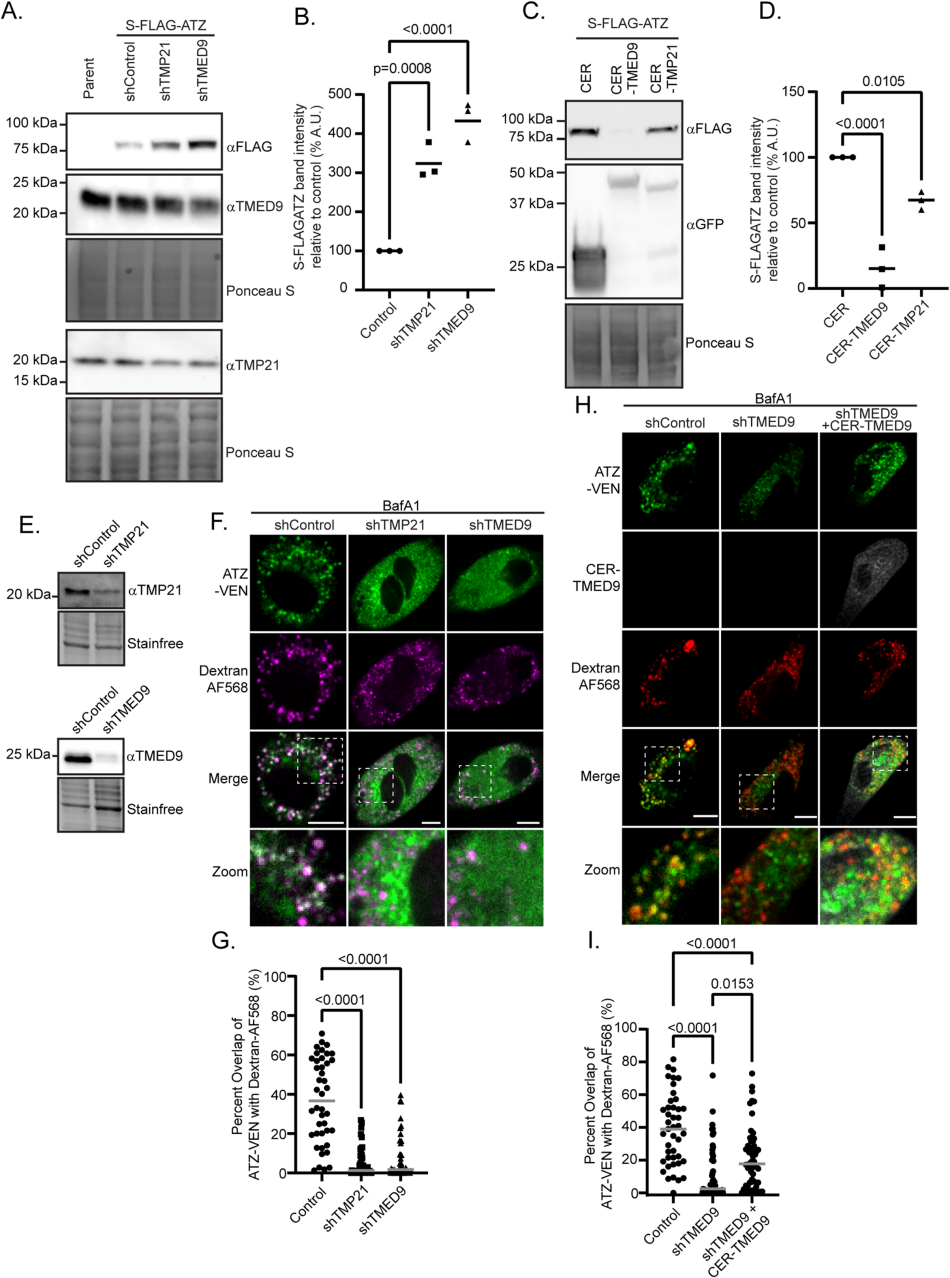
The p24-family is essential for ATZ degradation via ERLAD. (A) Cell lysates from N2a cells cotransfected with S-FLAG-ATZ and either shControl, shTMP21, or shTMED9 were analyzed by immunoblot. Membranes were stained with Ponceau S to visualize total protein and probed for FLAG, TMP21, and TMED9. Similar expression patterns for TMP21, TMED9, and FLAG proteins were obtained in each replicate experiment. (B) ATZ band intensity (FLAG) was measured and plotted for samples expressing shTMP21 (*n* = 3, mean = 325.7 ± 45%) or shTMED9 (*n* = 3, mean = 434 ± 51.7%) relative to ATZ in shControl samples, line represents mean. *P* values were calculated with a one-way ANOVA with Dunnett’s test. (C) Cell lysates from N2a cells cotransfected with S-FLAG-ATZ and either CER, CER-TMP21, or CER-TMED9 were analyzed by immunoblot. Membranes were stained with Ponceau S to visualize total protein and probed for FLAG and GFP. Similar expression patterns for FLAG and GFP were obtained in each replicate experiment. (D) ATZ band intensity (FLAG) was measured and plotted for cells samples expressing CER-TMP21 (*n* = 3, mean = 67 ± 7%) or CER-TMED9 (*n* = 3, mean = 16 ± 15%) relative to ATZ in cells expressing CER alone, line represents mean. *P* values were calculated with a one-way ANOVA with Dunnett’s test. (E) Cell lysates from NRK cells expressing shControl, shTMP21, or shTMED9 were analyzed by immunoblot. Membranes were stained with Ponceau for total protein and probed for TMP21 or TMED9. (F) NRK cells stably expressing ATZ-VEN and either shControl, shTMP21, or shTMED9 were loaded with dextran (dextran-AF568) before being treated with bafilomycin A1 overnight (see Supplemental Videos 9-11). Cells were subsequently fixed and imaged for colocalization analysis. Arrowheads point to ATZ colocalizing with dextran. (G) The Mander’s overlap coefficient for VEN-ATZ with dextran was measured for individual cells. The percentage of ATZ-VEN overlapping dextran is plotted for cells expressing shControl (*n* = 42, mean = 37 ± 21%), shTMP21 (*n* = 41, mean = 6 ± 8%), or shTMED9 (*n* = 42, mean = 8 ± 11%), line represents median. *P* values were calculated with a Mann-Whitney test. (H) NRK cells stably expressing ATZ-VEN and either shControl or shTMED9 were loaded with dextran (Dextran-AF568) before being treated with bafilomycin A1 overnight. A third group of cells stably expressing shTMED9 were transfected with CER-TMED9, loaded with dextran, and treated with bafilomycin A1 overnight. Cells were subsequently fixed and imaged for colocalization analysis. (I) The Mander’s overlap coefficient for ATZ-VEN with dextran was measured for individual cells. The percentage of ATZ-VEN overlapping dextran is plotted for individual cells expressing shControl (*n* = 43, mean = 39 ± 22%), shTMED9 (*n* = 59, mean = 11 ± 16%), or shTMED9+CER-TMED9 (*n* = 56, mean = 21 ± 18%), line represents median. *P* values were calculated with a Kruskal-Wallis test. All experiments were completed three or more times. Similar results were obtained in each replicate experiment. The experiments shown in panels (A–I) were repeated in three or more biological replicates. Scale bars = 10 μm.

The p24-family members TMP21 and TMED9 were previously shown to play an essential role in the lysosomal degradation of misfolding mutants of PrP and mucin-1, respectively (Satpute-Krishnan *et al.*, 2014; Dvela-Levitt *et al.*, 2019), and in this study we have found an association of ATZ with TMP21 and TMED9 (Figures 1F, 3C). Thus, we asked whether loss of either TMP21 or TMED9 affected ATZ degradation. To test this, we transfected N2a cells with S-FLAG-ATZ and either shControl, a shRNA targeting TMP21 (shTMP21), or a shRNA targeting TMED9 (shTMED9) and probed cell lysates for FLAG, TMED9, and TMP21. Despite multiple attempts, we could only isolate viable populations of transfected cells expressing shRNA at levels that lead to partial knockdown of TMP21 or TMED9. This may be due to the requirement of TMP21 and TMED9 in maintaining each other’s and other essential p24-family member’s stability and viability in cell culture (Blomen *et al.*, 2015; Tashima *et al.*, 2022). However, depletion of either TMP21 or TMED9 significantly increased intracellular ATZ levels (Figure 4, A and B). Conversely, we found that overexpression of either TMED9 or TMP21 significantly reduced intracellular S-FLAG-ATZ levels in N2a cells compared with cells expressing a control plasmid (Figure 4, C and D).

**Figure:** Movie S9 Control shRNA has no effect on ERLAD. A representative video of an NRK cell co‐expressing shControl and ATZ‐VEN (green). Cells were loaded with Dextran‐AF568 (magenta) and imaged over 12 hours after treatment with bafilomycin A1.

**Figure:** Movie S10 Loss of TMP21 blocks ERLAD of ATZ. A representative video of an NRK cell co‐expressing shTMP21 and ATZ‐VEN (green). Cells were loaded with Detran‐AF568 (magenta) and imaged over 12 hours after treatment with bafilomycin A1.

**Figure:** Movie S11 Loss of TMED9 blocks ERLAD of ATZ. A representative video of an NRK cell co‐expressing shTMED9 and ATZ‐VEN (green). Cells were loaded with Detran‐AF568 (magenta) and imaged over 12 hours after treatment with bafilomycin A1.

We next sought to confirm whether loss of either TMED9 or TMP21 reduced the accumulation of ATZ-VEN in lysosomes. To test this, we generated NRK cells stably expressing ATZ-VEN and either shControl, shTMP21, or shTMED9 (Figure 4E). To mark lysosomes, we preloaded ATZ-VEN cells with fluorescently conjugated dextran beads and then treated the cells with bafilomycin A1. We imaged these cells for 12 h and measured the colocalization of ATZ with dextran-marked lysosomes (Figure 4F; Supplemental videos 9–11). After 12 h, we found that a significantly larger fraction of the total ATZ-VEN fluorescence colocalized with dextran-marked vesicles in control cells (mean = 37 ± 21%, *n* = 42) compared with cells expressing shTMP21 (mean = 6 ± 8%, *n* = 41) or shTMED9 (mean = 8 ± 11%, *n* = 42; Figure 4G). Together, these data support the hypothesis that TMP21 and TMED9 play key roles in regulating ATZ degradation.

Given the obvious impact of TMED9 on regulating ERLAD of ATZ observed in Figure 4, A–G, we asked whether expression of a shRNA-resistant CER-TMED9 construct could rescue lysosomal trafficking of ATZ in shTMED9-expressing cells. Starting with NRK cells that stably expressed ATZ-VEN and shTMED9 with or without transient transfection of CER-TMED9, we again marked the lysosomes by preloading the cells with dextran. In control cells treated overnight with bafilomycin A1, 39 ± 22% (*n* = 43) of ATZ-VEN colocalized with dextran, while only 11 ± 16% (*n* = 59) of the ATZ-VEN colocalized with lysosomes in shTMED9 cells. Overexpressing shRNA-resistant CER-TMED9 in the shTMED9 cells increased colocalization of ATZ-VEN with lysosomes to 21 ± 18% (*n* = 56). Although the rescue was partial, colocalization of ATZ-VEN with lysosomes was significantly increased in the shRNA cells that were rescued with CER-TMED9 (Figure 4, H and I).

## DISCUSSION

Over the past 5 years, several studies have elucidated pathways by which misfolded, aggregated secretory proteins that are recalcitrant to ERAD are cleared from the ER to lysosomes. These pathways are collectively designated as “ERLAD” (Fregno *et al.*, 2018; De Leonibus *et al.*, 2019; Fregno and Molinari, 2019; Wilkinson, 2020; Molinari, 2021). Newly uncovered proteins involved in these ERLAD pathways include the ER-phagy receptor FAM134B for the clearance of ATZ (Fregno *et al.*, 2018) and the COPII coat protein SEC24C for the clearance of misfolded variants of proarginine-vasopressin, proopiomelanocortin, and the proinsulin mutant *Akita* (Cui *et al.*, 2019; Parashar *et al.*, 2021). Additionally, the Ferro-Novick group demonstrated that SEC24C is required for the delivery of FAM134B from the ER to lysosomes and introduced the concept of ER-phagy exit sites, coining the term “ERPHES” (Cui *et al.*, 2019), thus exposing an important connection between ER-phagy receptors and the COPII coat. During the final step of ATZ delivery to lysosomes, the interaction between FAM134B’s LC3-interaction region and lysosome-localized LC3-II was shown to be critical (Fregno *et al.*, 2018). However, the factors that mediate the linkage between misfolded proteins, SEC24C and FAM134B at the ER-localized ERLAD exit sites remained unidentified.

The propensity of p24-family members to associate with misfolded GPI-APs or the MUC1 frameshift mutant protein within the lumen of the ER (Satpute-Krishnan *et al.*, 2014; Dvela-Levitt *et al.*, 2019; Zavodszky and Hegde, 2019) together with the p24-family members’ well-characterized cytosolic COPII binding motifs for direct binding to SEC24 (Dominguez *et al.*, 1998; Tang *et al.*, 1999; Miller *et al.*, 2003; Bonnon *et al.*, 2010; Castillon *et al.*, 2011; D’Arcangelo *et al.*, 2015; Roberts and Satpute-Krishnan, 2022) equips the p24-family with well-placed features to connect ATZ and SEC24C at COPII-coated ERLAD exit sites. Thus, we set out to test whether SEC24C and the p24-family members are involved in the ERLAD of ATZ.

We show here for the first time that the expression of SEC24C, TMP21, and TMED9 are each required for the degradation of ATZ through ERLAD (Figures 2 and 4). Additional key findings presented here include that (a) ATZ-VEN copurifies with detectably more TMP21, TMED9, calnexin and FAM134B than AAT-VEN does (Figure 1F; Supplemental Figure 1C), (b) CER-tagged p24-family members, TMP21, TMED9 and TMED2, each pull down ATZ, FAM134B, and SEC24C (Figure 3C), (c) TMP21, TMED9, and ATZ cotraffic from the ER to lysosomes, and are blocked together at ER exit sites in BFA and nocodazole (BFA+Noc) treated cells, or are redirected together to an ER-derived compartment in 3-methyladenine treated cells (Figures 1 and 3). Together, these findings not only fit with but also build upon the model for ERLAD of ATZ presented by Fregno *et al.* 2018. To explain the packaging of ATZ into ERLAD vesicles, we propose a model wherein calnexin releases ATZ and FAM134B to the p24-family members, which recruit SEC24C along with the COPII coat proteins to promote ERLAD vesicle formation (Supplemental Figure S3).

Our study, which reveals a new requisite role for TMP21 and TMED9 in the ERLAD of ATZ, sets the stage for future investigations into the detailed mechanisms by which p24-family of proteins coordinate the clearance of misfolded ER proteins through the ERLAD pathway. These findings raise many new questions and future directions relating to ATZ, the p24-family and SEC24C including the following. (1) What is the polymerization or aggregation state of the ATZ substrate that assembles with the p24-family for ERLAD? (2) Are associations between ATZ and p24-family members dependent on the presentation of a misfolding domain? Are these associations direct or through a chaperone intermediary? Are these features shared by other p24-family interacting misfolding proteins (e.g., PrP* [Satpute-Krishnan *et al.*, 2014; Zavodszky and Hegde, 2019] and mucin-1 mutants [Dvela-Levitt *et al.*, 2019])? (3) Considering that previously published large scale affinity-purification mass spectrometry datasets of FAM134B coimmunoprecipitates included TMP21, TMED9 and p24 (Grumati *et al.*, 2017; Gonzalez *et al.*, 2023), what is the nature of the association between FAM134B and the p24-family members? (4) In addition to assembling with ATZ, FAM134B and SEC24C at the ER, do the p24-family members play roles in subsequent steps of the ERLAD pathway? For example, TMP21 and TMED9 have been shown to directly bind with the C-terminal tail of syntaxin 17 (Muppirala *et al.*, 2011), and syntaxin 17 is a SNARE required for the transfer of ATZ from the ER lumen into lysosomes (Fregno *et al.*, 2018). (5) Detection of Sec23-mCH at the sites where ATZ colocalize with SEC24C in BFA+Noc treated cells (Figure 3, A and B) suggest that other COPII coat constituents may assemble at ER-exit sites for ERLAD. What are the differences between the COPII coat constituents that assemble at ER-exit sites destined for ERLAD versus Golgi? Finally, additional experiments are required to test our proposed model for how TMP21 or TMED9 coordinate the trafficking of ATZ along the ERLAD pathway (Supplemental Figure S3).

Challenges to answering these questions through depletion studies include that individual p24-family members function together as heterooligomers and stabilize each other (Belden and Barlowe, 1996;Emery *et al.*, 2000; Jenne *et al.*, 2002; Fujita *et al.*, 2011; Tashima *et al.*, 2022), TMP21 and other p24-family members are essential for cell culture and early embryonic viability (Denzel *et al.*, 2000; Jerome-Majewska *et al.*, 2010; Blomen *et al.*, 2015), and that the p24-family members play diverse and fundamental roles in organizing the early secretory pathway at the structural and molecular (i.e., protein and lipid) levels (Schimmoller *et al.*, 1995; Barr *et al.*, 2001; Belden and Barlowe, 2001; Emery *et al.*, 2003; Mitrovic *et al.*, 2008; Strating *et al.*, 2009; D’Arcangelo *et al.*, 2015; Anwar *et al.*, 2022). Precise replacements of endogenous p24-family members with truncation and domain swap mutants may help to dissect the interactions between ERLAD substrates and p24-family members with minimal perturbations to cell viability, and the organization and molecular composition across the overall endomembrane system.

## MATERIALS AND METHODS

Request a protocol through *Bio-protocol*.

### Cell culture conditions and small molecules

Mouse N2a cells were obtained from ATCC (ATCC CCL-131). The NRK cells used here were provided by Jennifer Lippincott-Schwartz lab and first characterized in their classic secretory pathway studies (Lippincott-Schwartz *et al.*, 1989, 1990; Donaldson *et al.*, 1990, 1991), and more recently in studies examining autophagy (Hailey *et al.*, 2010), or degradation of misfolded proteins via ERAD and RESET (Satpute-Krishnan *et al.*, 2014).

Cells were incubated at 37°C and 5% CO_2_ in complete DMEM containing Dulbecco’s Modified Eagle Medium (DMEM, Corning 17-205-CV) containing 10% fetal bovine serum (FBS, Corning 35-011-CV), 1x L-glutamine (Corning, 25-005-CL). Cells used in experiments were regularly split every 2 or 3 d using 0.05% Trypsin/0.53 mM EDTA in HBSS (Corning 25-051-Cl), and never allowed to grow beyond 80% confluency. Cells were monitored for growth and confirmed to be a pure population using the standard tissue culture microscope, Leica DMi1 FOV20 Basic Stand outfitted with a 40X HI PLAN, 20X, 10X, and 5X objective and slider for phase contrast. N2a cells were confirmed to match the mixture of neuronal and amoeboid morphology shown in the image provided by ATCC for ATCC CCL-131, and NRK cells were confirmed to exhibit their adherent, epithelial morphology and to grow as monolayer. Before experiments, cells were monitored for bacterial or fungal contamination using the Leica DMi1 or for mycoplasma infection with the Mycostrip-Mycoplasma Detection Kit (Invivogen rep-mys-50) according to the manufacturer’s instructions.

For secretion studies, N2a cells were switched from complete medium to DMEM containing 1% FBS, 1x L-glutamine, and 1x penicillin/streptomycin for 4 h before medium was collected for analysis.

Cells were treated with chemical inhibitors in complete DMEM at the following concentrations: 2.5 uM brefeldin A (LC Laboratories B-8500), 1.25 ug/mL nocodazole (SCBT SC-3518B), 250 nM bafilomycin A1 (SCBT SC-201550B), 10 mM 3-methyladenine (Sigma M928).

### Plasmids

Sec23-mCherry was obtained from Addgene (Addgene plasmid # 166894, gift of Jennifer Lippincott-Schwartz).

CFP-PrP* is identical to YFP-PrP*, whose construction was described in detail (Satpute-Krishnan *et al.*, 2014), except that the YFP was replaced with CFP within the single Bsu361 restriction site.

For the following plasmids whose ORFs have not been described in detail elsewhere, we provide the ORF sequences and state the vector backbone.

SS(TMP21)-CER-TMP21: “SS” refers to signal sequence. The SS(TMP21)-GFP-TMP21 plasmid was generously gifted to us by Robert Blum. The construction of SS(TMP21)-GFP-TMP21 in the pEGFP-C1 (Clontech) plasmid backbone was described in detail previously (Blum *et al.*, 1999; Blum and Lepier, 2008). Starting with Blum’s SS(TMP21)-GFP-TMP21, we destroyed the AgeI site in the vector backbone that begins 13 nucleotides upstream from the start codon using site directed mutagenesis to make ACCGGT to AAGGGT. Removing the upstream Age1 site allowed us to use the AgeI and BsRGI sites in common between fluorescent proteins to replace the GFP cDNA sequence with CER. Upon ligation, the final open reading frame was confirmed through sequencing analysis to be as follows with TMP21 (rat) signal sequence and TMP21 (rat) ORF in all caps, and the linkers and CER in all lowercase:

ATG TCT GGT TTG TCT GGC CCA CCA GCC CGG CGC GGC CCT TTT CCG TTA GCG TTG CTG CTT TTG TTC CTG CTC GGC CCC AGA TTG GTC CTT GCC cta ccg gtc gcc acc atg gtg agc aag ggc gag gag ctg ttc acc ggg gtg gtg ccc atc ctg gtc gag ctg gac ggc gac gta aac ggc cac aag ttc agc gtg tcc ggc gag ggc gag ggc gat gcc acc tac ggc aag ctg acc ctg aag ctg atc tgc acc acc ggc aag ctg ccc gtg ccc tgg ccc acc ctc gtg acc acc ctg ggc tac ggc ctg cag tgc ttc gcc cgc tac ccc gac cac atg aag cag cac gac ttc ttc aag tcc gcc atg ccc gaa ggc tac gtc cag gag cgc acc atc ttc ttc aag gac gac ggc aac tac aag acc cgc gcc gag gtg aag ttc gag ggc gac acc ctg gtg aac cgc atc gag ctg aag ggc atc gac ttc aag gag gac ggc aac atc ctg ggg cac aag ctg gag tac aac tac aac agc cac aac gtc tat atc acc gcc gac aag cag aag aac ggc atc aag gcc aac ttc aag atc cgc cac aac atc gag gac ggc ggc gtg cag ctc gcc gac cac tac cag cag aac acc ccc atc ggc gac ggc ccc gtg ctg ctg ccc gac aac cac tac ctg agc tac cag tcc aag ctg agc aaa gac ccc aac gag aag cgc gat cac atg gtc ctg ctg gag ttc gtg acc gcc gcc ggg atc act ctc ggc atg gac gag ctg tac aag tcc gga ctc aga tct cga gct caa gct tcg AAT TCT ATC TCC TTC CAT CTA CCC GTG AAC TCT CGG AAG TGT CTC CGC GAG GAG ATC CAC AAA GAC TTG CTG GTT ACG GGC GCG TAC GAG ATC ACC GAC CAG TCT GGG GGC GCT GGC GGC CTG CGC ACC CAC CTC AAG ATC ACA GAT TCT GCT GGC CAT ATT CTG TAT GCC AAA GAG GAT GCA ACT AAA GGG AAG TTT GCC TTT ACC ACA GAA GAC TAT GAC ATG TTT GAA GTA TGC TTT GAG AGC AAG GGA ACA GGG CGG ATA CCT GAC CAA CTC GTG ATT CTA GAC ATG AAG CAT GGA GTA GAG GCG AAA AAT TAT GAA GAG ATC GCA AAA GTT GAG AAA CTC AAA CCA CTG GAG GTG GAG CTA CGG CGG CTC GAG GAC CTT TCA GAG TCT ATT GTT AAC GAC TTT GCC TAC ATG AAG AAG CGG GAA GAG GAG ATG AGG GAC ACC AAC GAG TCC ACA AAC ACC CGC GTC CTG TAC TTC AGC ATC TTT TCC ATG TTC TGC CTC ATT GGA CTA GCC ACC TGG CAG GTC TTC TAC CTG CGT CGC TTC TTC AAG GCC AAG AAG TTG ATA GAG TAA

ATZ-VEN, AAT-VEN and AAT-CER: Starting with AAT-GFP, described previously (Granell *et al.*, 2008), which was generously gifted by Giulia Baldini, we PCR amplified the AAT cDNA with primers encoding the BamHI and EcoRI cut sites, digested and inserted it into the pVenus-N1 (Addgene plasmid # 54640, gifted by Mike Davidson) or pCerulean-N1 (Addgene plasmid # 54742, gifted by Mike Davidson) multicloning site. The difference between AAT and ATZ is the E366K mutation. Please note that E366K is referenced as E342K in some publications to indicate the mutation in the context of the polypeptide sequence after signal sequence processing. ATZ-VEN was generated from AAT-VEN by using the NEB Site-Directed Mutagenesis kit (NEB E0554S) to change GAG to AAG in the codon at nucleotide number 1043, which is underlined in the below ORF. The final open reading frame for AAT-VEN was confirmed through sequencing analysis to be as follows with AAT in all capital letters and linker and VEN in lower case.

ATG CCG TCT TCT GTC TCG TGG GGC ATC CTC CTG CTG GCA GGC CTG TGC TGC CTG GTC CCT GTC TCC CTG GCT GAG GAC CCC CAG GGA GAT GCT GCC CAG AAG ACA GAT ACA TCC CAC CAT GAT CAG GAT CAC CCA ACC TTC AAC AAG ATC ACC CCC AAC CTG GCT GAG TTC GCC TTC AGC CTA TAC CGC CAG CTG GCA CAC CAG TCC AAC AGC ACC AAT ATC TTC TTC TCC CCA GTG AGC ATC GCT ACA GCC TTT GCA ATG CTC TCC CTG GGG ACC AAG GCT GAC ACT CAC GAT GAA ATC CTG GAG GGC CTG AAT TTC AAC CTC ACG GAG ATT CCG GAG GCT CAG ATC CAT GAA GGC TTC CAG GAA CTC CTC CGT ACC CTC AAC CAG CCA GAC AGC CAG CTC CAG CTG ACC ACC GGC AAT GGC CTG TTC CTC AGC GAG GGC CTG AAG CTA GTG GAT AAG TTT TTG GAG GAT GTT AAA AAG TTG TAC CAC TCA GAA GCC TTC ACT GTC AAC TTC GGG GAC ACC GAA GAG GCC AAG AAA CAG ATC AAC GAT TAC GTG GAG AAG GGT ACT CAA GGG AAA ATT GTG GAT TTG GTC AAG GAG CTT GAC AGA GAC ACA GTT TTT GCT CTG GTG AAT TAC ATC TTC TTT AAA GGC AAA TGG GAG AGA CCC TTT GAA GTC AAG GAC ACC GAG GAA GAG GAC TTC CAC GTG GAC CAG GTG ACC ACC GTG AAG GTG CCT ATG ATG AAG CGT TTA GGC ATG TTT AAC ATC CAG CAC TGT AAG AAG CTG TCC AGC TGG GTG CTG CTG ATG AAA TAC CTG GGC AAT GCC ACC GCC ATC TTC TTC CTG CCT GAT GAG GGG AAA CTA CAG CAC CTG GAA AAT GAA CTC ACC CAC GAT ATC ATC ACC AAG TTC CTG GAA AAT GAA GAC AGA AGG TCT GCC AGC TTA CAT TTA CCC AAA CTG TCC ATT ACT GGA ACC TAT GAT CTG AAG AGC GTC CTG GGT CAA CTG GGC ATC ACT AAG GTC TTC AGC AAT GGG GCT GAC CTC TCC GGG GTC ACA GAG GAG GCA CCC CTG AAG CTC TCC AAG GCC GTG CAT AAG GCT GTG CTG ACC ATC GAC GAG AAA GGG ACT GAA GCT GCT GGG GCC ATG TTT TTA GAG GCC ATA CCC ATG TCT ATC CCC CCC GAG GTC AAG TTC AAC AAA CCC TTT GTC TTC TTA ATG ATT GAA CAA AAT ACC AAG TCT CCC CTC TTC ATG GGA AAA GTG GTG AAT CCC ACC CAA AAA gga tcc acc ggt cgc cac atg gtg agc aag ggc gag gag ctg ttc acc ggg gtg gtg ccc atc ctg gtc gag ctg gac ggc gac gta aac ggc cac aag ttc agc gtg tcc ggc gag ggc gag ggc gat gcc acc tac ggc aag ctg acc ctg aag ctg atc tgc acc acc ggc aag ctg ccc gtg ccc tgg ccc acc ctc gtg acc acc ctg ggc tac ggc ctg cag tgc ttc gcc cgc tac ccc gac cac atg aag cag cac gac ttc ttc aag tcc gcc atg ccc gaa ggc tac gtc cag gag cgc acc atc ttc ttc aag gac gac ggc aac tac aag acc cgc gcc gag gtg aag ttc gag ggc gac acc ctg gtg aac cgc atc gag ctg aag ggc atc gac ttc aag gag gac ggc aac atc ctg ggg cac aag ctg gag tac aac tac aac agc cac aac gtc tat atc acc gcc gac aag cag aag aac ggc atc aag gcc aac ttc aag atc cgc cac aac atc gag gac ggc ggc gtg cag ctc gcc gac cac tac cag cag aac acc ccc atc ggc gac ggc ccc gtg ctg ctg ccc gac aac cac tac ctg agc tac cag tcc gcc ctg agc aaa gac ccc aac gag aag cgc gat cac atg gtc ctg ctg gag ttc gtg acc gcc gcc ggg atc act ctc ggc atg gac gag ctg tac aag taa

LAMP1-mCH: LAMP1-mCherry was a generous gift from George Patterson, National Institutes of Health, Bethesda, MD, USA. It is LAMP1 with a C-terminal mCherry tag that was constructed as described for PA-GFP-lgp120 in a pEGFP-N1 Clontech vector backbone (Patterson and Lippincott-Schwartz, 2002), but with mCherry exchanged for the PA-GFP. The open reading frame was verified by sequencing to be as follows with Lamp1 (rat) ORF in capital letters and linker and mCherry in lower case: ATG GCG GCC CCG GGC GCC CGG CGG CCG CTG CTC CTG TTG CTG CTG GCA GGC CTT GCA CAC AGC GCC CCA GCA CTG TTC GAG GTG AAA GAC AAC AAC GGC ACA GCG TGT ATA ATG GCC AGC TTC TCT GCC TCC TTT CTG ACC ACC TAT GAG GCT GGA CAT GTT TCT AAG GTC TCG AAT ATG ACC CTG CCA GCC TCT GCA GAA GTC CTG AAG AAT AGC AGC TCT TGT GGT GAA AAG AAT GCT TCT GAG CCC ACC CTC GCA ATC ACC TTT GGA GAA GGA TAT TTA CTG AAA CTC ACC TTC ACA AAA AAC ACA ACA CGT TAC AGT GTC CAG CAC ATG TAT TTC ACA TAT AAC CTG TCA GAC ACA CAA TTC TTT CCC AAT GCC AGC TCC AAA GGG CCC GAC ACT GTG GAT TCC ACA ACT GAC ATC AAG GCA GAC ATC AAC AAA ACA TAC CGA TGT GTC AGC GAC ATC AGG GTC TAC ATG AAG AAT GTG ACC ATT GTG CTC TGG GAC GCT ACT ATC CAG GCC TAC CTG CCG AGT AGC AAC TTC AGC AAG GAA GAG ACA CGC TGC CCA CAG GAT CAA CCT TCC CCA ACT ACT GGG CCA CCC AGC CCC TCA CCA CCA CTT GTG CCC ACA AAC CCC AGT GTG TCC AAG TAC AAT GTG ACT GGT GAC AAT GGA ACC TGC CTG CTG GCC TCT ATG GCA CTG CAA CTC AAC ATC ACC TAC ATG AAG AAG GAC AAC ACG ACT GTG ACC AGA GCA TTC AAC ATC AAC CCA AGT GAC AAA TAT AGT GGG ACT TGC GGT GCC CAG TTG GTG ACC CTG AAG GTG GGG AAC AAG AGC AGA GTC CTG GAG CTG CAG TTT GGG ATG AAT GCC ACT TCT AGC CTG TTT TTC CTG CAA GGA GTT CAG TTG AAC ATG ACT CTT CCT GAT GCC ATA GAG CCC ACG TTC AGC ACC TCC AAC TAT TCC CTG AAA GCT CTT CAG GCC AGT GTC GGC AAC TCA TAC AAG TGC AAC AGT GAG GAG CAC ATC TTT GTC AGC AAG GCG CTC GCC CTC AAT GTC TTC AGC GTG CAA GTC CAG GCT TTC AGG GTA GAA AGT GAC AGG TTT GGG TCT GTG GAA GAG TGT GTA CAG GAC GGT AAC AAC ATG CTG ATC CCC ATT GCT GTG GGC GGG GCC CTG GCA GGG CTG GTC CTC ATC GTC CTC ATC GCC TAC CTC ATC GGC AGG AAG AGG AGT CAC GCG GGC TAT CAG ACC ATC tcg gaa ttc ggc tcc acc ggc tcc acc ggc tcc acc ggc gcg gat cca ccg gtc gcc acc atg gtg agc aag ggc gag gag gat aac atg gcc atc atc aag gag ttc atg cgc ttc aag gtg cac atg gag ggc tcc gtg aac ggc cac gag ttc gag atc gag ggc gag ggc gag ggc cgc ccc tac gag ggc acc cag acc gcc aag ctg aag gtg acc aag ggt ggc ccc ctg ccc ttc gcc tgg gac atc ctg tcc cct cag ttc atg tac ggc tcc aag gcc tac gtg aag cac ccc gcc gac atc ccc gac tac ttg aag ctg tcc ttc ccc gag ggc ttc aag tgg gag cgc gtg atg aac ttc gag gac ggc ggc gtg gtg acc gtg acc cag gac tcc tcc ctg cag gac ggc gag ttc atc tac aag gtg aag ctg cgc ggc acc aac ttc ccc tcc gac ggc ccc gta atg cag aag aag acc atg ggc tgg gag gcc tcc tcc gag cgg atg tac ccc gag gac ggc gcc ctg aag ggc gag atc aag cag agg ctg aag ctg aag gac ggc ggc cac tac gac gct gag gtc aag acc acc tac aag gcc aag aag ccc gtg cag ctg ccc ggc gcc tac aac gtc aac atc aag ttg gac atc acc tcc cac aac gag gac tac acc atc gtg gaa cag tac gaa cgc gcc gag ggc cgc cac tcc acc ggc ggc atg gac gag ctg tac aag taa

SS(lactase)-CER-TMED9: A series of plasmids were cloned using the ss(lactase)-CER backbone from the SS(lactase)-CER-Thy1 whose construction was described in detail in our previous work (Cheatham *et al.*, 2023). “SS(lactase)” refers to rabbit lactase-phlorizin hydrolase signal sequence. “Mature domain” refers to the ORF that encodes the processed type I transmembrane protein after the N-terminal signal sequence has been cleaved by the signal peptidase. Starting with SS(lactase)-CER-Thy1 (Cheatham *et al.*, 2023), the Thy1 mature domain was replace with the mature domain of TMED9, which was PCR amplified using the TMED9 (human) cDNA (Genscript NM_017510.6) as the template. For assembly we used NEBuilder (NEB E5520S). The final ORF was verified to be the following through sequencing analysis with SS(lactase) in all capital letters, followed by linker and CER in lowercase, followed by TMED9 mature domain in capital letters:

ATG GAG CTC TTT TGG AGT ATA GTC TTT ACT GTC CTC CTG AGT TTC TCC TGC CGG GGG TCA GAC TGG GAA TCT CTG CAG TCG ACG GTA CCG CGG GCC CGG GAT CCA CCG GTC GCC ACC atg gtg agc aag ggc gag gag ctg ttc acc ggg gtg gtg ccc atc ctg gtc gag ctg gac ggc gac gta aac ggc cac aag ttc agc gtg tcc ggc gag ggc gag ggc gat gcc acc tac ggc aag ctg acc ctg aag ttc atc tgc acc acc ggc aag ctg ccc gtg ccc tgg ccc acc ctc gtg acc acc ctg acc tgg ggc gtg cag tgc ttc gcc cgc tac ccc gac cac atg aag cag cac gac ttc ttc aag tcc gcc atg ccc gaa ggc tac gtc cag gag cgc acc atc ttc ttc aag gac gac ggc aac tac aag acc cgc gcc gag gtg aag ttc gag ggc gac acc ctg gtg aac cgc atc gag ctg aag ggc atc gac ttc aag gag gac ggc aac atc ctg ggg cac aag ctg gag tac aac gcc atc agc gac aac gtc tat atc acc gcc gac aag cag aag aac ggc atc aag gcc aac ttc aag atc cgc cac aac atc gag gac ggc agc gtg cag ctc gcc gac cac tac cag cag aac acc ccc atc ggc gac ggc ccc gtg ctg ctg ccc gac aac cac tac ctg agc acc cag tcc aag ctg agc aaa gac ccc aac gag aag cgc gat cac atg gtc ctg ctg gag ttc gtg acc gcc gcc ggg atc act ctc ggc atg gac gag ctg tac aag gca gga ggc agc CTC TAC TTT CAC ATC GGA GAG ACG GAG AAG AAG TGC TTT ATT GAG GAG ATC CCG GAC GAG ACC ATG GTC ATA GGA AAC TAC CGG ACG CAG CTG TAT GAC AAG CAG CGG GAG GAG TAC CAG CCG GCC ACC CCG GGG CTT GGC ATG TTT GTG GAG GTG AAG GAC CCA GAG GAC AAG GTC ATC CTG GCC CGG CAG TAT GGC TCC GAG GGC AGG TTC ACT TTC ACT TCC CAT ACC CCT GGT GAG CAC CAG ATC TGT CTT CAC TCC AAT TCC ACC AAG TTC TCC CTC TTT GCT GGA GGC ATG CTG AGA GTT CAC CTG GAC ATC CAG GTA GGT GAA CAT GCC AAT GAC TAT GCA GAA ATT GCT GCT AAA GAC AAG TTG AGT GAG TTG CAG CTA CGA GTG CGA CAG CTG GTG GAA CAA GTG GAG CAG ATC CAG AAA GAG CAG AAC TAC CAG CGG TGG CGA GAG GAG CGC TTC CGG CAG ACC AGT GAG AGC ACC AAC CAG CGG GTG CTG TGG TGG TCC ATT CTG CAG ACC CTC ATC CTC GTG GCC ATC GGT GTC TGG CAG ATG CGG CAC CTC AAG AGC TTC TTT GAA GCC AAG AAG CTT GTG TAG.

SS(lactase)-VEN-AAT and SS(lactase)-VEN-ATZ: These were generated by starting with SS(lactase)-CER-TMED9 (described above) and replacing CER with VEN through BsrGI and AgeI restriction cut sites, and then replacing the TMED9 mature domain with AAT and ATZ mature domains cloned from the AAT-VEN and ATZ-VEN (described above) using NEBuilder system (NEB E5520S), with the only the difference between AAT and ATZ is at the codon “GAG”, which is switched to “AAG” at nucleotide number 1043 underlined in the below ORF. The final ORFs were verified to be the following through sequencing analysis with SS (lactase) in all capital letters, followed by linker and VEN in lowercase, followed by the AAT mature domain in capital letters:

ATG GAG CTC TTT TGG AGT ATA GTC TTT ACT GTC CTC CTG AGT TTC TCC TGC CGG GGG TCA GAC TGG GAA TCT CTG CAG TCG ACG GTA CCG CGG GCC CGG GAT CCA CCG GTC GCC ACC atg gtg agc aag ggc gag gag ctg ttc acc ggg gtg gtg ccc atc ctg gtc gag ctg gac ggc gac gta aac ggc cac aag ttc agc gtg ggc gag ggc gag ggc gat gcc acc tac ggc aag ctg acc ctg aag ctg atc tgc acc acc ggc aag ctg ccc gtg ccc tgg ccc acc ctc gtg acc acc ctg ggc tac ggc ctg cag tgc ttc gcc cgc tac ccc gac cac atg aag cag cac gac ttc ttc aag tcc gcc atg ccc gaa ggc tac gtc cag gag cgc acc atc ttc ttc aag gac gac ggc aac tac aag acc cgc gcc gag gtg aag ttc gag ggc gac acc ctg gtg aac cgc atc gag ctg aag ggc atc gac ttc aag gag gac ggc aac atc ctg ggg cac aag ctg gag tac aac tac aac agc cac aac gtc tat atc acc gcc gac aag cag aag aac ggc atc aag gcc aac ttc aag atc cgc cac aac atc gag gac ggc ggc gtg cag ctc gcc gac cac tac cag cag aac acc ccc atc ggc gac ggc ccc gtg ctg ctg ccc gac aac cac tac ctg agc tac cag tcc aag ctg agc aaa gac ccc aac gag aag cgc gat cac atg gtc ctg ctg gag ttc gtg acc gcc gcc ggg atc act ctc ggc atg gac gag ctg tac aag gca gga ggc agc GAG GAC CCC CAG GGA GAT GCT GCC CAG AAG ACA GAT ACA TCC CAC CAT GAT CAG GAT CAC CCA ACC TTC AAC AAG ATC ACC CCC AAC CTG GCT GAG TTC GCC TTC AGC CTA TAC CGC CAG CTG GCA CAC CAG TCC AAC AGC ACC AAT ATC TTC TTC TCC CCA GTG AGC ATC GCT ACA GCC TTT GCA ATG CTC TCC CTG GGG ACC AAG GCT GAC ACT CAC GAT GAA ATC CTG GAG GGC CTG AAT TTC AAC CTC ACG GAG ATT CCG GAG GCT CAG ATC CAT GAA GGC TTC CAG GAA CTC CTC CGT ACC CTC AAC CAG CCA GAC AGC CAG CTC CAG CTG ACC ACC GGC AAT GGC CTG TTC CTC AGC GAG GGC CTG AAG CTA GTG GAT AAG TTT TTG GAG GAT GTT AAA AAG TTG TAC CAC TCA GAA GCC TTC ACT GTC AAC TTC GGG GAC ACC GAA GAG GCC AAG AAA CAG ATC AAC GAT TAC GTG GAG AAG GGT ACT CAA GGG AAA ATT GTG GAT TTG GTC AAG GAG CTT GAC AGA GAC ACA GTT TTT GCT CTG GTG AAT TAC ATC TTC TTT AAA GGC AAA TGG GAG AGA CCC TTT GAA GTC AAG GAC ACC GAG GAA GAG GAC TTC CAC GTG GAC CAG GTG ACC ACC GTG AAG GTG CCT ATG ATG AAG CGT TTA GGC ATG TTT AAC ATC CAG CAC TGT AAG AAG CTG TCC AGC TGG GTG CTG CTG ATG AAA TAC CTG GGC AAT GCC ACC GCC ATC TTC TTC CTG CCT GAT GAG GGG AAA CTA CAG CAC CTG GAA AAT GAA CTC ACC CAC GAT ATC ATC ACC AAG TTC CTG GAA AAT GAA GAC AGA AGG TCT GCC AGC TTA CAT TTA CCC AAA CTG TCC ATT ACT GGA ACC TAT GAT CTG AAG AGC GTC CTG GGT CAA CTG GGC ATC ACT AAG GTC TTC AGC AAT GGG GCT GAC CTC TCC GGG GTC ACA GAG GAG GCA CCC CTG AAG CTC TCC AAG GCC GTG CAT AAG GCT GTG CTG ACC ATC GAC GAG AAA GGG ACT GAA GCT GCT GGG GCC ATG TTT TTA GAG GCC ATA CCC ATG TCT ATC CCC CCC GAG GTC AAG TTC AAC AAA CCC TTT GTC TTC TTA ATG ATT GAA CAA AAT ACC AAG TCT CCC CTC TTC ATG GGA AAA GTG GTG AAT CCC ACC CAA AAA TGA.

SS (lactase)-S-FLAG-AAT or SS (lactase)-S-FLAG-ATZ: “S-FLAG” refers to the SNAP-FLAG, which was derived from SNAP-FLAG-CD59 (obtained from Addgene, plasmid # 50374, deposited by Reika Watanabe) by PCR amplification. “FLAG” is synonymous with the amino acid sequence DYKDDDDK. SS (lactase)-S-FLAG-AAT/ATZ are identical to the SS (lactase)-VEN-AAT/ATZ plasmids annotated above with the following exception. Using the NEBuilder system (NEB E5520S), we replaced the VEN and linker portion with the “S-FLAG” and linker sequence that is listed below, and verified the construct by sequencing analysis.

atg gac aaa gac tgc gaa atg aag cgc acc acc ctg gat agc cct ctg ggc aag ctg gaa ctg tct ggg tgc gaa cag ggc cac cgt atc atc ttc ctg ggc aaa gga aca tct gcc gcc gac gcc gtg gaa gtg cct gcc cca gcc gcc gtg ctg ggc gga cca gag cca ctg atg cag gcc acc gcc tgg ctc aac gcc tac ttt cac cag cct gag gcc atc gag gag ttc cct gtg cca gcc ctg cac cac cca gtg ttc cag cag gag agc ttt acc cgc cag gtg ctg tgg aaa ctg ctg aaa gtg gtg aag ttc gga gag gtc atc agc tac agc cac ctg gcc gcc ctg gcc ggc aat ccc gcc gcc acc gcc gcc gtg aaa acc gcc ctg agc gga aat ccc gtg ccc att ctg atc ccc tgc cac cgg gtg gtg cag ggc gac ctg gac gtg ggg ggc tac gag ggc ggg ctc gcc gtg aaa gag tgg ctg ctg gcc cac gag ggc cac aga ctg ggc aag cct ggg ctg ggt gcc ctg cag gac tac aag gac gac gat gac aag gca gga ggc agc

The targeting shRNAs shTMED9 (Sigma TRCN0000302434), shTMP21 (Sigma TRCN0000348406), shSEC24C (Sigma TRCN0000100582) and nontargeting control shRNAs (Sigma TRCN0000278237; Addgene 162011) were used in knockdown experiments.

### Transfections

N2a and NRK cells were transfected using Lipofectamine 3000 (Invitrogen L3000001) or PolyJet (SignaGen SL100688) according to the manufacturer’s suggestions.

### Microscopy

Cells were seeded onto a #1.5 coverslip-bottom tissue culture dish. For live-cell experiments, cells were maintained in complete DMEM cell culture medium at 37°C with 5% CO_2_. Imaging experiments were performed at 50–80% confluency of stably-transfected cells or 48 h after transfection, unless otherwise indicated. All images were acquired using a Nikon inverted spinning disk confocal microscope equipped with a Yokogawa CSU-X1 Spinning Disk, EMCCD camera, 60x Plan Apo 1.40 NA oil / 0.13 mm WD, Tokai live-cell incubator, and 445 nm, 514, 561, and 647 nm laser lines. Focus drift was prevented through the Perfect Focus system.

### Image analysis

Colocalization analysis: Colocalization was measured in FiJi (Schindelin *et al.*, 2012) using the JaCoP plugin. First, Z-stack images were cropped to include a single cell. A 1-pixel median filter was applied to cropped images and a consistent threshold was set for each set of channels. In knockdown studies, the top 2% of pixel intensities was selected as a threshold in each channel; in ATZ-VEN/SEC23-mCH studies, 1% of pixel intensities was selected as a threshold in each channel using the last time point as a reference image; in ERGIC53 studies, thresholds were calculated using the Rényi entropy method for each image. Selected thresholds were used to analyze the Mander’s overlap coefficient in JaCoP (Bolte and Cordelieres, 2006).

### Immunoblot analysis

For immunoblot analysis of whole cell lysates, cells were briefly rinsed with phosphate-buffered saline (PBS) and lysed in harsh lysis buffer (100 mM NaCl, 25 mM HEPES pH 7.5, 2 mM MgCl_2_, 1% Triton X-100, 0.1% sodium dodecyl sulfate [SDS]). Protein lysate concentrations were then measured by Pierce Rapid Gold BCA Protein Assay Kit (Pierce A53225). Equal amounts of each sample were combined with 4xSB + 10 mM TCEP, which refers to 4X sample buffer (Bio-Rad 1610747) and the reducing agent, tris (2-carboxyethyl) phosphine (Sigma 646547). To perform the SDS–PAGE and western blot procedures, we used the Bio-Rad V3 Western Workflow, which includes the TGX Pre-Cast Stain-free gels and system, Trans-Blot Turbo Transfer System and ChemiDoc System. In the cases where we were unable to obtain TGX Pre-Cast Stain-free gels, due to COVID19 pandemic supply shortages, we used AdvanStain Ponceau stain (Advansta R-03021-D50) to detect total protein in the lysates. After we completed the Stain-free system, membranes were blocked with 5% nonfat dry milk in TBS + 0.1% Tween (TBST). Primary and HRP-conjugated secondary antibodies were incubated on a shaker in 5% nonfat dry milk in TBST and washed three times in TBST after each incubation. Membranes were submerged in Clarity (Bio-Rad 1705061) or Clarity Max enhanced chemiluminescence substrate (Bio-Rad 1705062), and chemiluminescence was imaged with a Bio-Rad ChemiDoc system. Band intensities were measured using the Image Lab software (Bio-Rad).

The following primary antibodies were used for immunoblot analysis: FLAG (GenScript A00187; 1:5000), GFP (homemade, 1:2500), TMP21 (homemade, 1:5000), TMED9 (Proteintech 21620-1-AP, 1:2500), calnexin (Enzo Life Science ADI-SPA-860-F, 1:2500), SEC24C (Invitrogen PA5-59101; 1:2000), FAM134B (Proteintech 21537-1-AP, 1:2500). Specificity of the primary antibodies were confirmed by comparing the band sizes with expected band sizes based on their corresponding specification sheets or the literature cited. For FLAG or GFP, controls included comparison of bands in transfected versus untransfected cells. For TMP21, TMED9 and SEC24C, additional controls included shRNA depletion. For FAM134B, additional controls included negative controls in the coimmunoprecipitation studies. Secondary antibodies were used: HRP-conjugated anti-rabbit (Cytiva NA934-1ML, 1:5000), HRP-conjugated anti-mouse (Invitrogen A28177, 1:5000).

### Coimmunoprecipitations

GFP pull-downs were performed 48 h after transient transfection of N2a cells that were cultured in 10-cm dishes to 60–80% confluency by the μMACS GFP Isolation Kit (Miltenyi Biotec 130-091-288) and μMACS DYKDDDDK (FLAG-tag) Isolation Kit (Miltenyi Biotec 130-101-591). All pull-downs were performed exactly according to the manufacturer’s recommendations, with one exception: a homemade lysis buffer, which was originally optimized to maintain protein–protein interactions with calnexin (Satpute-Krishnan *et al.*, 2014), comprising 1% CHAPS, 50 mM HEPES, pH 7.4, 100 mM NaCl, and 2 mM CaCl2 was used to lyse cells, incubate lysate with magnetic antibody-conjugated microbeads, and wash beads that were trapped on the magnetic column. The entire coimmunoprecipitation procedure before elution was performed in a 4°C cold room and on ice to stabilize interactions. Specificity of the GFP and FLAG antibodies were determined by including untransfected parental cell lines in our pull down studies.

### Immunofluorescence

For immunofluorescence studies, NRK cells were first washed with PBS and fixed with either 3:1 methanol:acetone solution at –20°C or 4% paraformaldehyde “PFA” (Electron Microscopy Science 15710) for 10 min. The samples fixed in PFA were permeabilized with 0.1% Triton X-100 in PBS for 15 min. Samples were blocked with UltraCruz blocking solution (Santa-Cruz SC-516214) for 1 h at room temperature. Samples were then probed for 1 to 1.5 h with primary antibody, rinsed with PBS and probed with a conjugated secondary antibody. Samples were then rinsed with PBS and imaged in PBS.

Primary antibodies used for immunofluorescence studies included rabbit anti-ERGIC53 (Proteintech 13364-1-AP, 1:500), and rabbit anti-SEC24C (Novus NBP1-81550, 1:200 or Invitrogen PA5-59101, 1:100). Specificity of immunofluorescence was confirmed with secondary-only controls in which the primary antibody was not included.

For experiments in which lysosomes were marked with Alexa Fluor 568-conjugated dextran (Invitrogen D22912), cells were incubated with 0.1 mg/ml dextran in complete media for 1 to 2 h. Cells were then rinsed and media was replaced. At the completion of the experiment, cells were fixed with 1% glutaraldehyde (Electron Microscopy Science 16010) + 3% PFA to preserve the fluorescence Alexa Fluor 568-conjugated dextran and imaged in PBS.

### Statistics

Statistical analyses were performed and graphed using Prism 9 (GraphPad). Statistical significance was calculated using a Mann-Whitney test or unpaired *t* test for two sample analyses or analysis of variance (ANOVA) for more than one sample. A Dunnett’s post-hoc test was used for one-way analyses and a Kruskal-Wallis test was used for two-way analyses as indicated. Where indicated, error is presented as SD from the mean.

## Supplementary Material



## Abbreviations used:

AAT: Alpha-1-antitrypsin
ATZ: Alpha-1-antitrypsin Z mutant
BFA: Brefeldin A
CER: Cerulean
COPII: Coat protein complex II
CFP: Cyan Fluorescent Protein
C-terminal: Carboxyl-terminal
DMEM: Dulbecco’s Modified Eagle Medium
ER: Endoplasmic Reticulum
ERAD: ER-Associated Degradation
ERLAD: ER-to-lysosome-associated-degradation
FLAG: DYKDDDDK amino acid sequence tag referred to as “FLAG”
GPI: Glycosylphosphatidylinositol
GPI-AP: Glycosylphosphatidylinositol-anchored protein
GFP: Green Fluorescent Protein
mCH: mCherry
PBS: Phosphate-buffered saline
PQC: Protein Quality Control
PrP*: Misfolded prion protein, PrP C179A
NRK: Normal Rat Kidney
N2a: Neuro-2a, mouse neuroblastoma
Noc: Nocodazole
N-terminal: Amino-terminal
RESET: Rapid ER Stress-induced Export
SNARE: Soluble N-ethylmaleimide-sensitive factor Attachment protein Receptors
S-FLAG: SNAP-FLAG
shRNA: short hairpin RNA
shControl: Control shRNA
shSEC24C: SEC24C shRNA
shTMP21: TMP21 shRNA
shTMED9: TMED9 shRNA
STX17: Syntaxin 17
SS(lactase): Rabbit phlorizin-hydrolase lactase signal sequence
VEN: Venus
YFP: Yellow Fluorescent Protein
