# Supplementary material for: The p24-family and COPII subunit SEC24C facilitate the clearance of alpha1-antitrypsin Z from the endoplasmic reticulum to lysosomes
Source: Mol Biol Cell. 2024 Feb 7;35(3):ar45. doi: 10.1091/mbc.E23-06-0257 (PMC10916869; doi:10.1091/mbc.E23-06-0257)
Supplement: Supplementary file 12 [file mbc-35-ar45-s001.pdf]

Supplementary Materials

Molecular Biology of the Cell

Roberts *et al*.

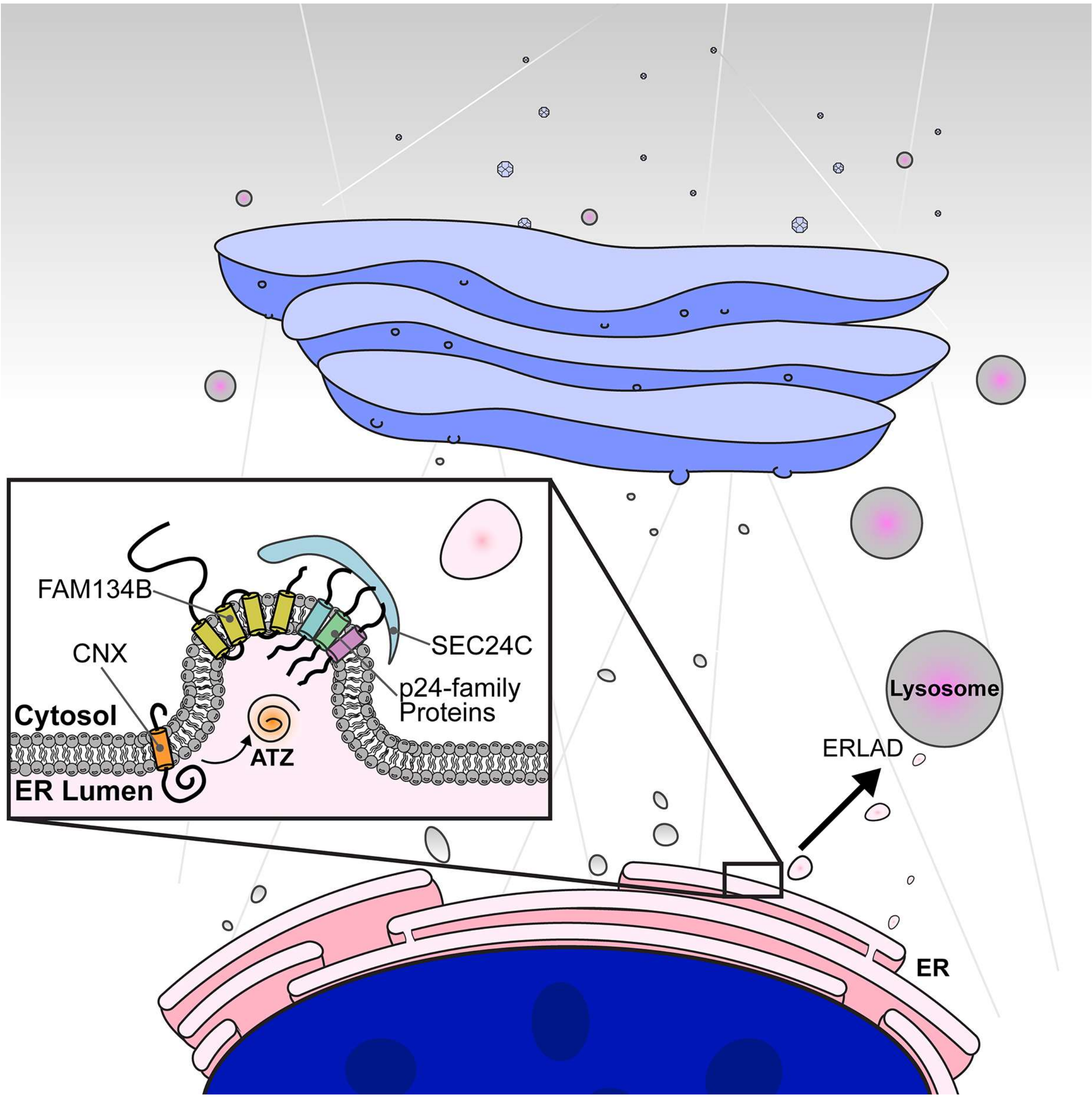

Supplementary figure 1. ATZ binds to TMP21 but does not undergo RESET. (A) Western blots depicting GFP-column purifications from lysates of untransfected (parent) N2a cells or

N2a cells stably transfected with YFP-PrP*, AAT-Ven or ATZ-Ven. Input, “I’ and eluate, ”E”. Red arrowhead indicates the expected molecular weight for ATZ-Ven and AAT-Ven, while blue asterisk lines up with YFP-PrP*. (B-C) CFP-PrP* traffics from the ER to the Golgi within 30m of thapsigargin (TG) treatment, while ATZ-VEN remains in the ER. Scale bar, 10um. (B) Time-lapse imaging of NRK cells co-expressing ATZ-Ven (pseudocolored green) and CFP-PrP* (pseudocolored red) after the addition of TG. (C) Immediately upon 30 minutes of thapsigargin treatment (“+TG 30m”), the same cell that is shown in panel B. was fixed and stained for the Golgi marker, GM130. Immunofluorescence of GM130 is pseudocolored blue. Scale bar = 10 μM.

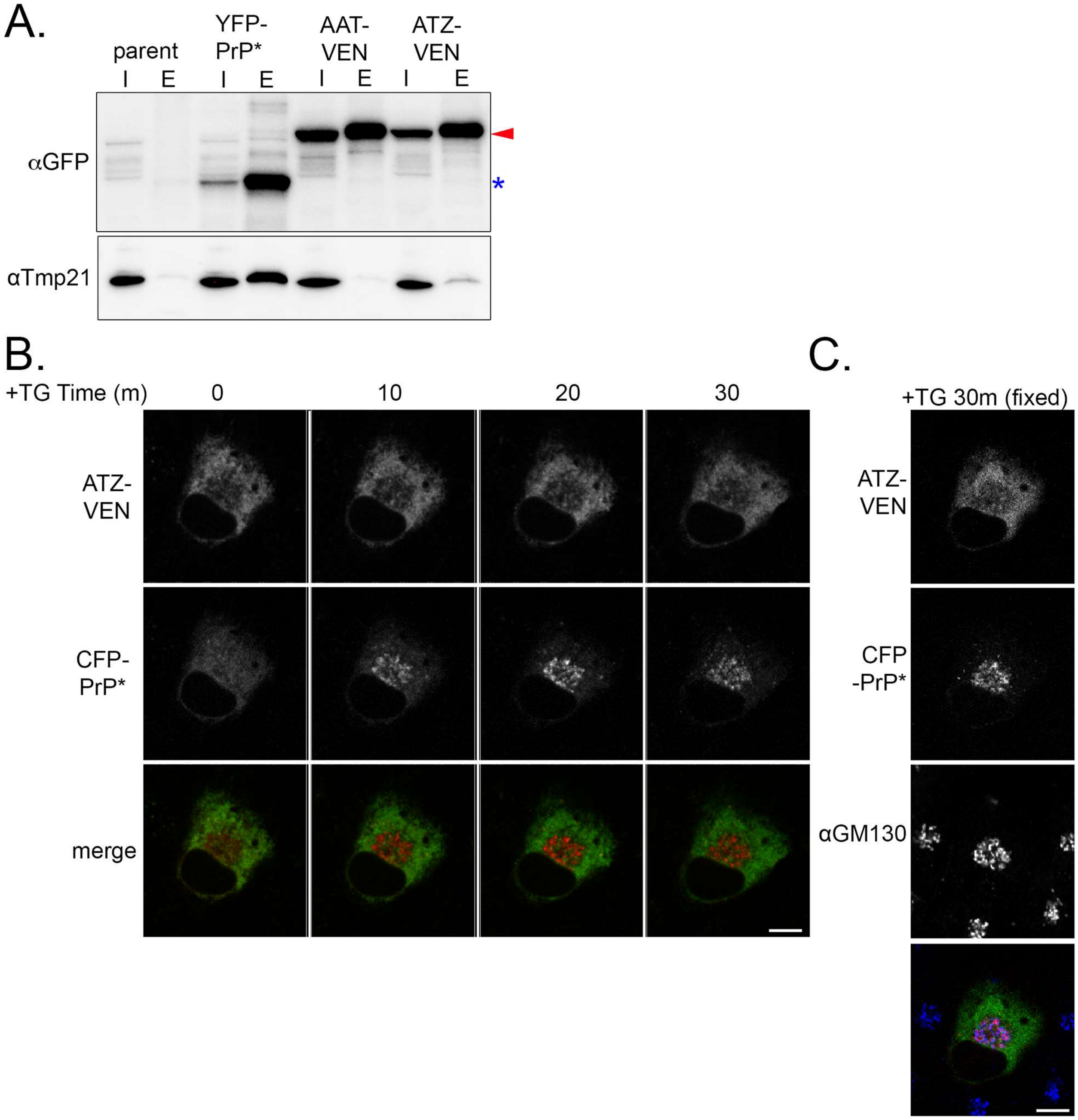

Supplementary figure 2. ATZ and TMP21 traffic to the lysosomes together in HeLa cells. HeLa cells were co-transfected with ATZ-VEN, CER-TMP21, and LAMP1-mCH and imaged over 12 hours after treatment with bafilomycin A1 (see Supplemental

Video 7). Arrows indicate sites of ATZ-VEN overlapping LAMP1-mCH Scale = 10 μm.

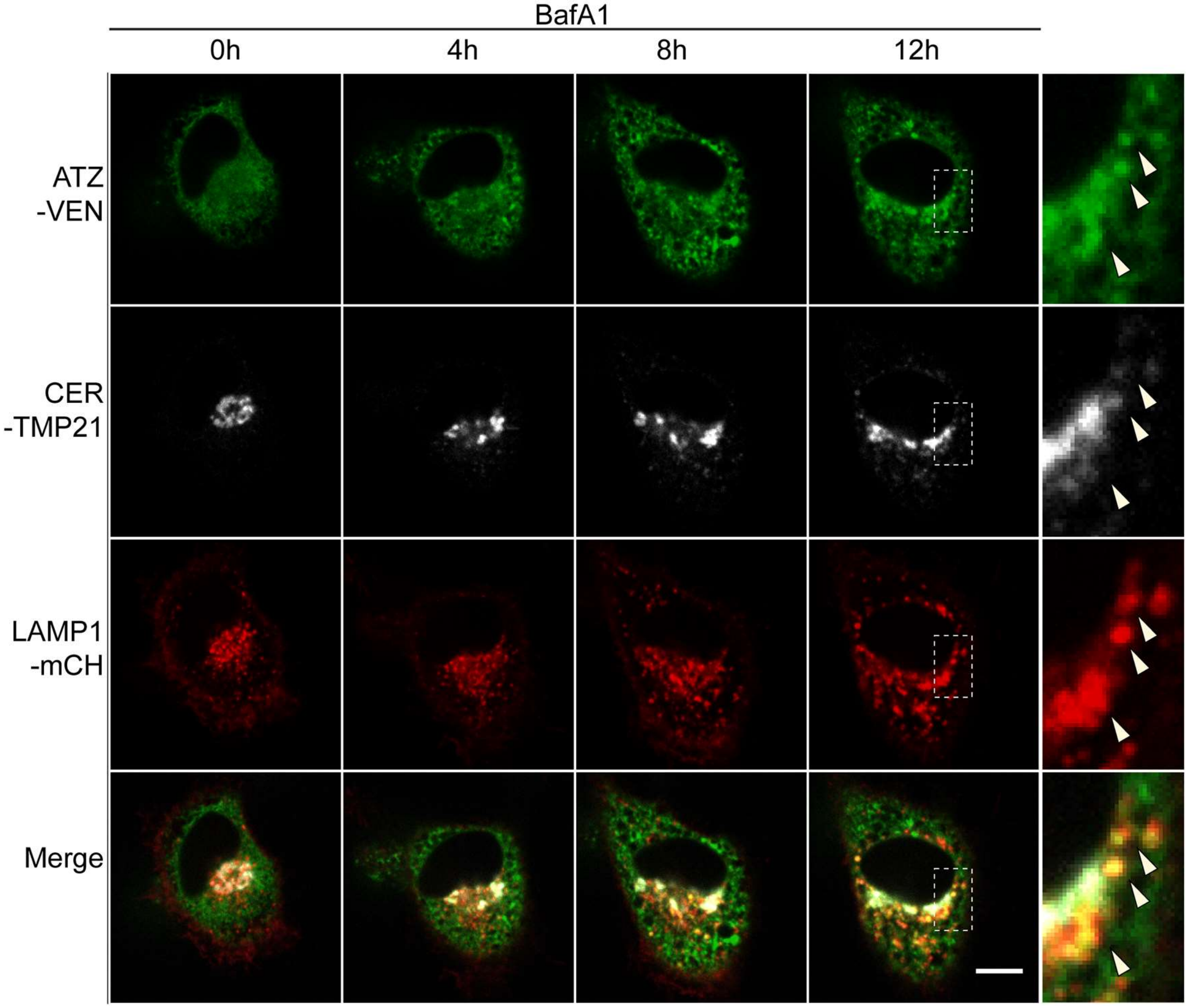

Supplementary Figure S3. A theoretical model for the role of the p24-family proteins in ERLAD. During ERLAD, calnexin (CNX) identifies and ushers misfolded ATZ into a subset of ER exit sites populated by FAM134B, TMP21, and TMED9. At these sites, TMP21 and TMED9 engage both FAM134B and ATZ, as well as the COPII subunit SEC24C. SEC24C recruitment promotes the formation of ER derived ERLAD vesicles which will traffic to and fuse with lysosomes.
